# Global Lactylome Reveals Lactylation‐Dependent Mechanisms Underlying CXC Motif Chemokine Ligand 12 Expression in Pulmonary Endothelium During Acute Respiratory Distress Syndrome

**DOI:** 10.1002/mco2.70344

**Published:** 2025-08-29

**Authors:** Xu Liu, Haofei Wang, Weijie Ni, Xuecheng Dong, Mingzhu Zheng, Wei Chang

**Affiliations:** ^1^ Jiangsu Provincial Key Laboratory of Critical Care Medicine. Department of Critical Care Medicine Zhongda Hospital, School of Medicine, Southeast University Nanjing Jiangsu China; ^2^ Institute of Nephrology Zhongda Hospital, School of Medicine, Southeast University Nanjing Jiangsu China; ^3^ Department of Respiratory Medicine Zhongda Hospital of Southeast University Nanjing Jiangsu China; ^4^ Department of Pathogenic Biology and Immunology School of Medicine, Jiangsu Provincial Key Laboratory of Critical Care Medicine, Southeast University Nanjing Jiangsu China

**Keywords:** ARDS, CXCL12, endothelium, Eno1, lactylation

## Abstract

Acute respiratory distress syndrome (ARDS) is a life‐threatening condition affecting millions of people worldwide. The severity of ARDS is associated with the dysfunction of pulmonary endothelial cells (PECs). Metabolic reprogramming is characterized by enhanced glycolysis and lactate accumulation, which play a critical role in this process. Here, we showed that lactate levels in the lungs of patients with ARDS were associated with disease severity and prognosis. Lactate promoted PEC dysfunction and drove experimental ARDS progression via lysine lactylation (Klac), a recently described posttranslational modification. Suppression of lactate‐induced lactylation mitigated the development of ARDS and inhibited the release of chemokines, particularly CXC motif chemokine ligand 12 (CXCL12), from PECs. Through quantitative lactylome analysis, we identified hyperlactylation at K193 of Enolase 1 (Eno1), a glycolytic enzyme with RNA‐binding capacity, as a previously unknown mechanism promoting CXCL12 production in PECs. Under homeostatic conditions, Eno1 could bind and inhibit the translation of CXCL12 mRNA, whereas increased glycolysis and accumulated lactate drove K193 hyperlactylation of Eno1 to release CXCL12 mRNA for accelerated translation. In addition, K193 hyperlactylation enhanced Eno1 enzymatic activity, further amplifying glycolysis. These findings establish Klac as a link between glycolytic reprogramming and PEC dysfunction, offering a new therapeutic target for ARDS.

## Graphical abstract

In quiescent PECs, Eno1 binds to the 3′ UTR of CXCL12 mRNA to prevent translation. Following activation, lactate is accumulated and then mediates an increase in lysine lactylation. Eno1, a substrate in this modification, releases bound CXCL12 mRNA upon lactylation to promote translation. Meanwhile, the activity of Eno1 as a glycolytic enzyme increases, which is required for the activated PECs to mediate inflammation.

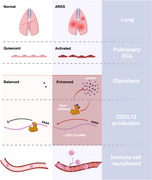

## 1 Introduction

Acute respiratory distress syndrome (ARDS) is a critical global health challenge, with a mortality rate of approximately 50% in moderate to severe degrees [1, 2, 3]. Despite its severity, effective treatments for established ARDS remain limited, largely due to an incomplete understanding of its etiology and pathogenesis.

Pulmonary endothelial cells (PECs) constitute over 30% of the lung cellular population and are integral to the gas exchange function of the pulmonary alveolus [4]. The roles of PECs in ARDS are often underestimated. However, growing evidence highlights the critical importance of PECs in maintaining lung tissue homeostasis and ARDS pathogenesis [5, 6]. PECs form a highly dynamic barrier that regulates immune cell recruitment and migration. When exposed to pathogenic factors, PECs become dysfunctional, releasing various chemokines that promote immune cell infiltration and amplify diffuse lung inflammation. Understanding the mechanisms underlying PEC‐mediated chemokine release may reveal novel therapeutic targets for ARDS.

PECs are metabolically active and rely primarily on glycolysis for energy production [7, 8]. In response to stimuli, PECs upregulate glycolysis as a compensatory mechanism to sustain adenosine triphosphate (ATP) synthesis [9]. Lactate, a by‐product of glycolysis, was traditionally considered a metabolic waste. However, this view has been increasingly challenged [10, 11]. Emerging evidence has implicated lactate in the pathogenesis of several diseases, including sepsis [12], cardiac fibrosis [13], and diabetic retinopathy [14]. Recently, Zhang et al. [15] identified a novel posttranslational modification (PTM), termed lysine lactylation (Klac), in which lactate adds lactyl groups to lysine residues, thereby influencing gene transcription and protein function. As a glycolysis‐responsive PTM [16], the role of lactylation in mediating PEC dysfunction during ARDS, particularly through glycolytic reprogramming, remains largely unexplored.

In the present study, we found that pulmonary lactate levels were strongly associated with ARDS severity and patient prognosis. Suppression of lactate‐derived lactylation using glycolysis inhibitors ameliorated experimental ARDS and inhibited the release of several chemokines from PECs, especially CXC motif chemokine ligand 12 (CXCL12). Mechanistically, we demonstrated that hyperlactylation of the glycolytic enzyme Enolase 1 (Eno1) at lysine 193 (K193), in particular, promoted CXCL12 production by modulating its RNA‐binding capacity. Inhibition of K193 lactylation on Eno1 significantly reduced the release of CXCL12 from PECs. These findings provide insights into the PEC dysfunction during ARDS, highlighting the importance of glycolysis reprogramming‐mediated lactylation in this process.

## 2 Results

### 2.1 Elevated Pulmonary Lactate Levels Are Associated With Poorer Survival in Patients With ARDS

**FIGURE 1:**
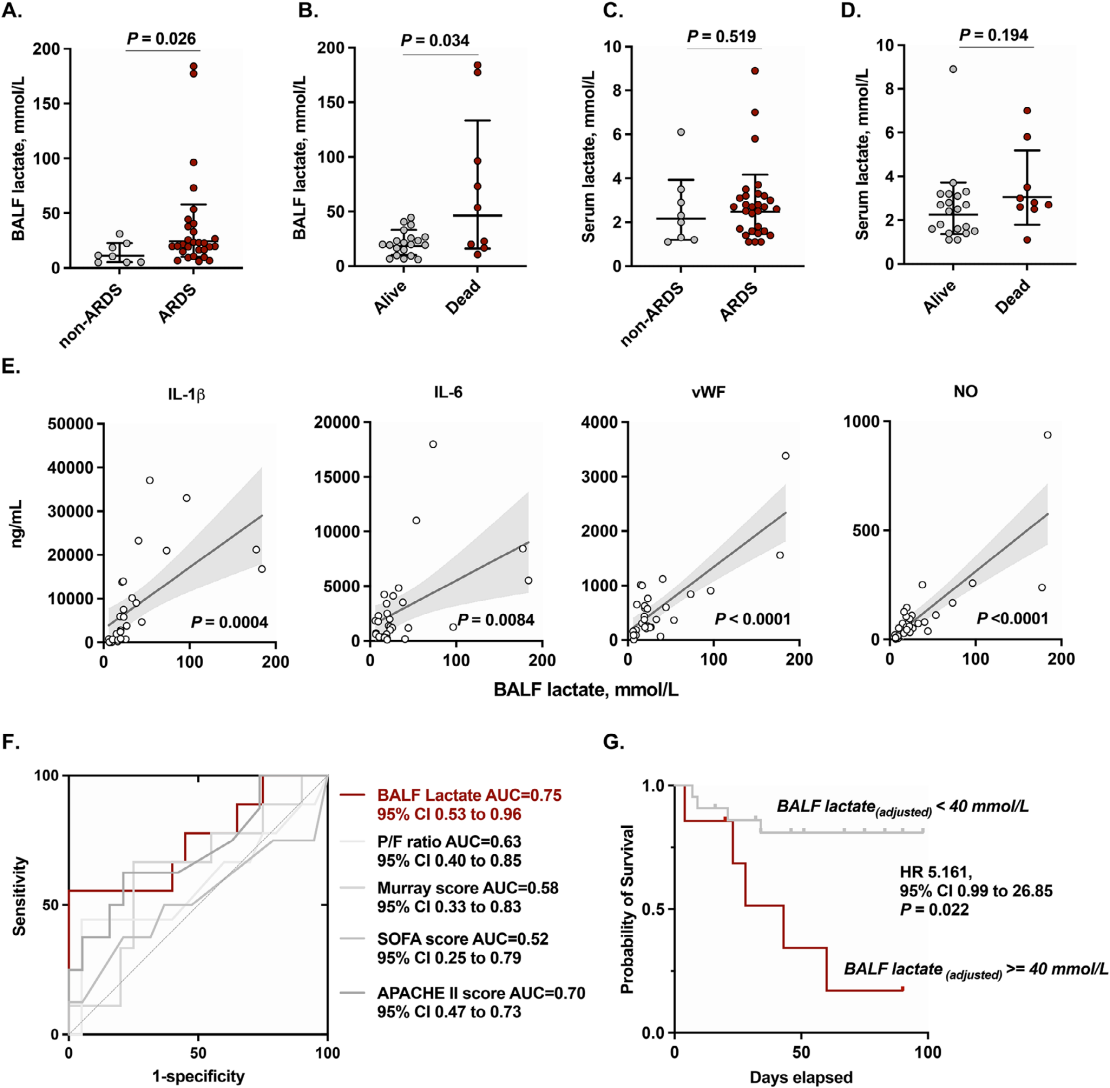
Elevated lactate levels in BALF were associated with worse survival for the patients with ARDS. (A) Lactate levels in BALF samples from non‐ARDS (*n* = 8) and ARDS patients (*n* = 29). (B) Lactate concentrations in BALF samples from survivors (*n* = 20) and non‐survivors (*n* = 9). (C) Lactate concentrations in serum samples from non‐ARDS (*n* = 8) and ARDS patients (*n* = 29). (D) Lactate concentrations in serum samples from survivors (*n* = 20) and non‐survivors (*n* = 9). (E) Correlation of BALF lactate levels with cytokines and endothelial dysfunction biomarkers in ARDS. (F) ROC curve of lactate levels, P/F ratio, Murray scores, SOFA scores, and APACHE II scores to predict ARDS 90‐day mortality in ARDS. (G) Kaplan–Meier survival curves of high BALF lactate (> 40 mmol/L) and low BALF lactate (< 40 mmol/L) were monitored up to 90 days after enrollment.

To investigate whether ARDS increases pulmonary lactate levels, we enrolled 29 patients with ARDS and 8 postoperative patients as controls. The demographic characteristics of patients were detailed in Table S1. Among the patients with ARDS, 20 survived, whereas 9 died at the end of the follow‐up (Table S2). The lactate concentrations in bronchoalveolar lavage fluid (BALF) were significantly higher in patients with ARDS than in non‐ARDS controls on the first day of ICU administration (Figure 1A). In addition, non‐survivors exhibited higher lactate concentrations than survivors (Figure 1B). By contrast, no significant differences in serum lactate concentrations were observed between patients with ARDS and non‐ARDS controls (Figure 1C). There were also no differences in serum lactate concentrations between survivors and non‐survivors (Figure 1D). Moreover, BALF lactate concentrations correlated significantly with the inflammatory mediators interleukin (IL)‐1β and IL‐6 and the endothelium activation biomarkers von Willebrand factor (vWF) and nitric oxide (NO) (Figure 1E).

To evaluate the association between BALF lactate levels and clinical outcomes, receiver operating characteristic (ROC) curves were conducted to predict 90‐day mortality using BALF lactate levels, Sequential Organ Failure Assessment (SOFA) scores, Acute Physiology and Chronic Health Evaluation II (APACHE II) scores, Murray scores, and the ratio of partial pressure of oxygen in arterial blood (PaO2) to the fraction of inspiratory oxygen concentration (FiO2) (P/F ratio) (Figure 1F). Among these metrics, BALF lactate exhibited the highest area under the ROC curve (AUC) at 0.75 (95% confidence interval [CI]: 0.53–0.96) for mortality prediction. An adjusted BALF lactate level cut‐off value of 40 mmol/L yielded a sensitivity of 0.56 (95% CI: 0.21–0.86) and specificity of 0.90 (95% CI: 0.68–0.99). The patients grouped according to the adjusted BALF lactate level by 40 mmol/L exhibited a significant difference in mortality by the Kaplan–Meier curve, with a hazard ratio of 5.161 (95% CI: 0.99–26.85) (Figure 1G).

Taken together, these findings suggest that elevated pulmonary lactate levels are a common feature of ARDS and are strongly correlated with increased mortality.

### 2.2 Inhibition of Lactate‐Derived Lactylation Ameliorates Experimental ARDS

**FIGURE 2:**
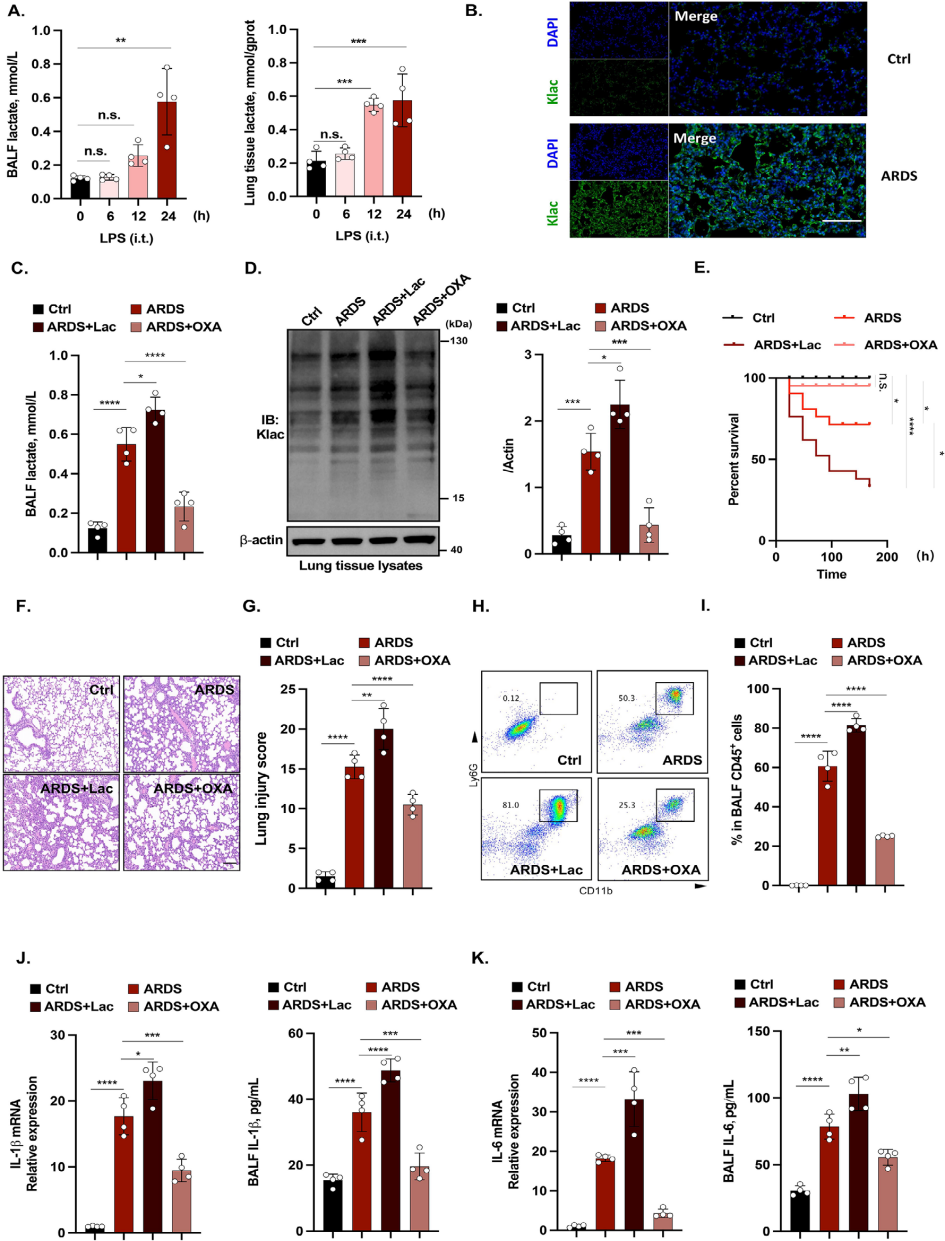
Inhibition of lactylation ameliorated ARDS progression. (A) Lactate levels in the BALF and parenchyma of the lungs of LPS‐induced ARDS mice increased over time (*n* = 3 per time point). (B) Representative immunofluorescence images of pan‐Klac in control and LPS‐induced ARDS mouse lungs. Scale bar, 50 µm. Experiments were performed three times. (C) Lactate concentration in the BALF of control mice, LPS‐induced ARDS mice, LPS‐induced ARDS mice+Lac, and LPS‐induced ARDS mice+OXA groups (*n* = 4 per group). (D) Western blotting and quantitative analysis of pan‐Klac in lung tissue lysates from control mice, LPS‐induced ARDS mice, LPS‐induced ARDS mice+Lac, and LPS‐induced ARDS mice+OXA groups. Experiments were performed four times. (E) Survival rates of the four groups (*n* = 21 per group). (F) HE staining and (G) quantification of the lung injury in control mice, LPS‐induced ARDS mice, LPS‐induced ARDS mice+Lac, and LPS‐induced ARDS mice+OXA groups (*n* = 4 per group). Scale bar, 50 µm. (H) Flow cytometry and (I) quantification of the neutrophil percentage in the BALF of the control mice, LPS‐induced ARDS mice, LPS‐induced ARDS mice+Lac, and LPS‐induced ARDS mice+OXA groups (*n* = 4 per group). (J–K) qPCR of inflammatory genes in lung tissue and their respective protein levels in BALF from control mice, LPS‐induced ARDS mice, LPS‐induced ARDS mice+Lac, and LPS‐induced ARDS mice+OXA groups (*n* = 4 per group). *p < 0.05, **p < 0.01, ***p < 0.001, ****p < 0.0001.

To further explore the relationship between lactate and ARDS, we used a lipopolysaccharide (LPS)‐induced experimental ARDS model. The lactate concentrations in the BALF and lung parenchyma increased progressively over time (Figure 2A). Given that Klac levels are predominantly determined by cellular lactate production, we hypothesized that lactylation is involved in ARDS progression. Consistent with this hypothesis, immunofluorescence staining revealed increased lactylation throughout the lung tissue in ARDS mice compared with control mice (Figure 2B). Consistently, the lactate concentrations in the BALF (Figure S1A,B) and the lactylation levels of lung tissues (Figure S1C,D) were also significantly increased in mice with cecal ligation and puncture (CLP) and hydrochloric acid (HCl) intratracheal instillation, indicating that ARDS is characterized by increased pulmonary lactylation levels despite varying etiologies.

Next, we examined whether lactate levels influence ARDS progression by modulating pulmonary lactylation. Additional lactate or sodium oxamate (OXA), a glycolysis inhibitor, was administered via intraperitoneal injection. Lactate administration significantly increased lung lactate concentrations, whereas OXA administration strongly inhibited lactate production in the lungs (Figure 2C). Consistently, additional lactate administration increased pulmonary lactylation levels in ARDS mice, whereas OXA reduced pulmonary lactylation levels (Figure 2D). Compared with the ARDS mice, mice in the ARDS+Lac group (ARDS mice treated with lactate) exhibited reduced survival rates (Figure 2E), more severe pulmonary damage (Figure 2F,G and Figure S2), and increased neutrophil recruitment (Figure 2H,I and Figure S3A). In contrast, mice in the ARDS+OXA group (ARDS mice treated with OXA) exhibited improved survival rates, decreased lung damage, and decreased neutrophil infiltration. In addition, the ARDS‐induced cytokines IL‐1β and IL‐6 were assessed across the groups. The IL‐1β and IL‐6 levels were higher in the ARDS+Lac group than in the ARDS group, whereas both cytokines were lower in the ARDS+OXA group (Figure 2J,K).

Collectively, our data support that elevated pulmonary lactylation induces ARDS progression, whereas pharmacological inhibition of lactylation suppresses ARDS.

### 2.3 Lactylation Modulates CXCL12 Release From PECs

**FIGURE 3:**
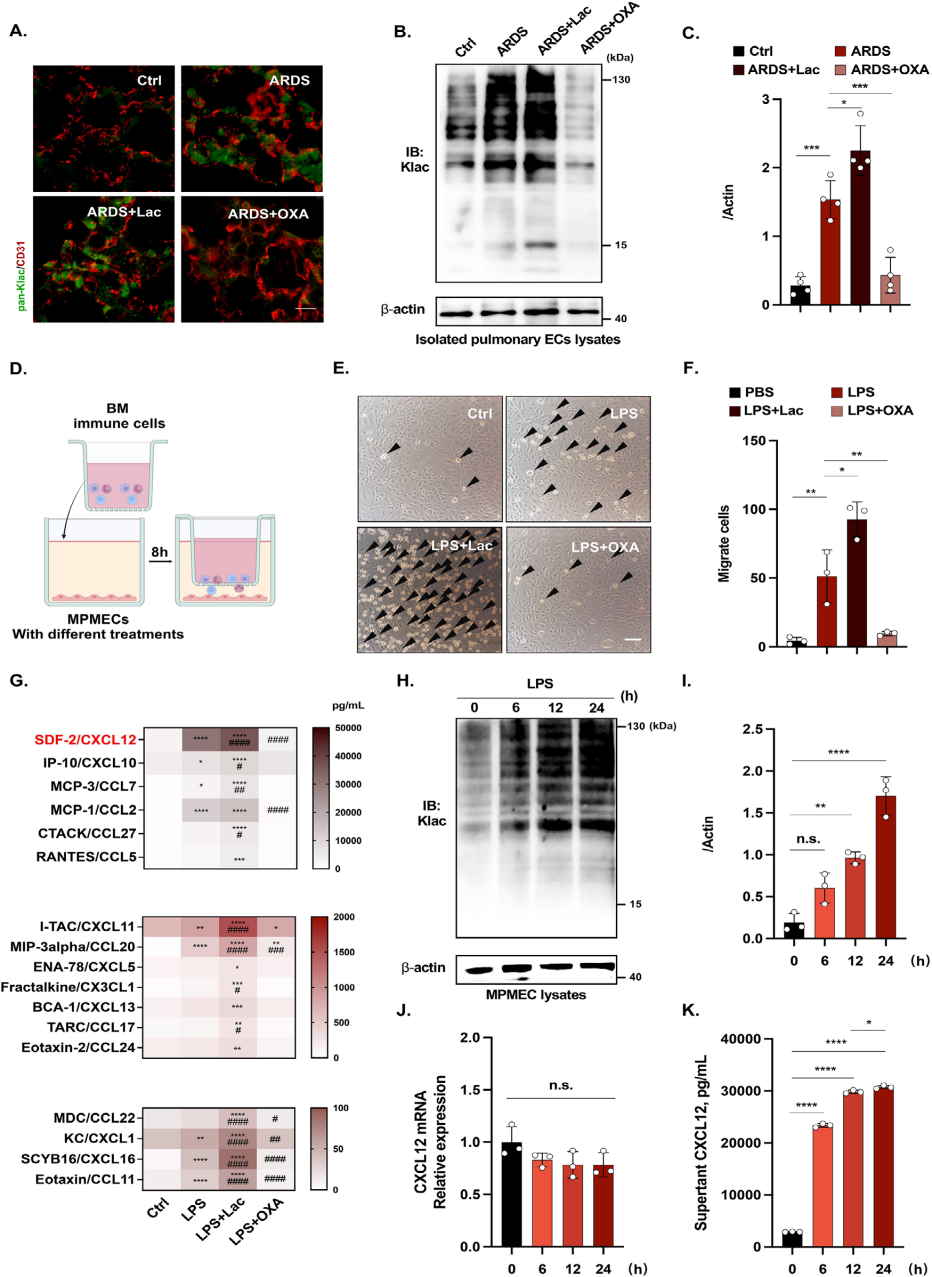
Lactylation affected the release of CXCL12 from PECs. (A) Representative immunofluorescence image of endothelial pan‐Klac in the lungs of control mice, LPS‐induced ARDS mice, LPS‐induced ARDS mice+Lac, and LPS‐induced ARDS mice+OXA groups (*n* = 4 per group). Scale bar, 10 µm. (B, C) Representative western blotting image and quantification of the pan‐Klac levels in primary PECs isolated from the lungs of control mice, LPS‐induced ARDS mice, LPS‐induced ARDS mice+Lac, and LPS‐induced ARDS mice+OXA groups (*n* = 4 per group). (D) Schematic representation of migration assay (created using Biorender.com). (E, F) The effect of modulating lactylation on MPMECs to attract BM immune cell migration was detected by transwell migration assay (*n* = 3 per group). (G) Luminex assays of multiplex chemokines in culture supernatants from the control, LPS, LPS+Lac, and LPS+OXA groups (*n* = 3 per group). (H, I) Western blotting analysis of pan‐Klac levels in MPMECs upon LPS stimulation at different time points. Experiments were performed three times. (J) qPCR analysis of CXCL12 mRNA expression in MPMECs after LPS challenge over time (*n* = 3). (K) CXCL12 production in the culture supernatants of MPMECs over time (*n* = 3). **p < 0.01, ***p < 0.005, ****p < 0.001. In G, *compared with the control group, *p < 0.05, **p < 0.01, ***p < 0.005, ****p < 0.001. ^#^Compared with the LPS group, ^#^ *p *< 0.05, ^##^ *p *< 0.01, ^###^ *p *< 0.005, ^####^ *p *< 0.001.

Given that PECs represent the largest lung cell population, we next investigated the potential link between lactylation and PEC dysfunction. Immunofluorescence staining (Figure 3A) combined with western blotting (Figure 3B,C and Figure S3B) revealed significantly elevated lactylation levels in the PECs of ARDS mice compared with the control mice. Additional lactate administration further increased PEC lactylation, whereas OXA treatment effectively reduced it.

To further elucidate the role of lactylation in PEC dysfunction, we conducted in vitro experiments using a mouse pulmonary microvascular endothelial cell line (MPMEC) previously established in our laboratory [17]. First, we tested the ability of PECs to attract immune cells by performing a Transwell migration assay through coculturing bone marrow (BM) immune cells and MPMECs subjected to different pretreatments (Figure 3D). Compared with the LPS group, MPMECs in the LPS+Lac group remarkably enhanced BM immune cell migration, whereas those in the LPS+OXA group attracted fewer BM immune cells (Figure 3E,F). Next, we measured the chemokine concentrations in the cell culture supernatant using Luminex Multiple Assays. LPS stimulation increased the production of nine chemokines, seven of which were significantly modulated by lactate and OXA treatment (Figure 3G). Among these, CXCL12, a potent neutrophil‐ and T cell‐attracting chemokine constitutively expressed in PECs (Figure S4A,B), was produced at substantially higher levels than the other six chemokines. We further confirmed that the CXCL12 concentrations in the BALF of ARDS mice changed with lactate levels (Figure S5A–E). Moreover, the CXCL12 concentrations correlated significantly with lactate levels in the BALF of patients with ARDS (Figure S6A,B). These findings suggest that lactylation may regulate the release of CXCL12 from PECs and promote ARDS progression.

To further explore the relationship between lactylation and CXCL12 production in PECs, we measured global lactylation levels and CXCL12 production in MPMECs following LPS stimulation in vitro. Lactylation levels in MPMECs progressively increased with LPS treatment (Figure 3H,I). Notably, although CXCL12 transcription remained unchanged (Figure 3J), its protein level increased in a time‐dependent manner (Figure 3K). We also observed that the CXCL12 production in HULEC‐5a, a human lung MPMEC, was correlated with cell lactylation levels (Figure S7A,B). These findings confirmed that lactylation acts as a regulator of CXCL12 production in PECs.

### 2.4 Global View of the Lactylome in PECs

**FIGURE 4:**
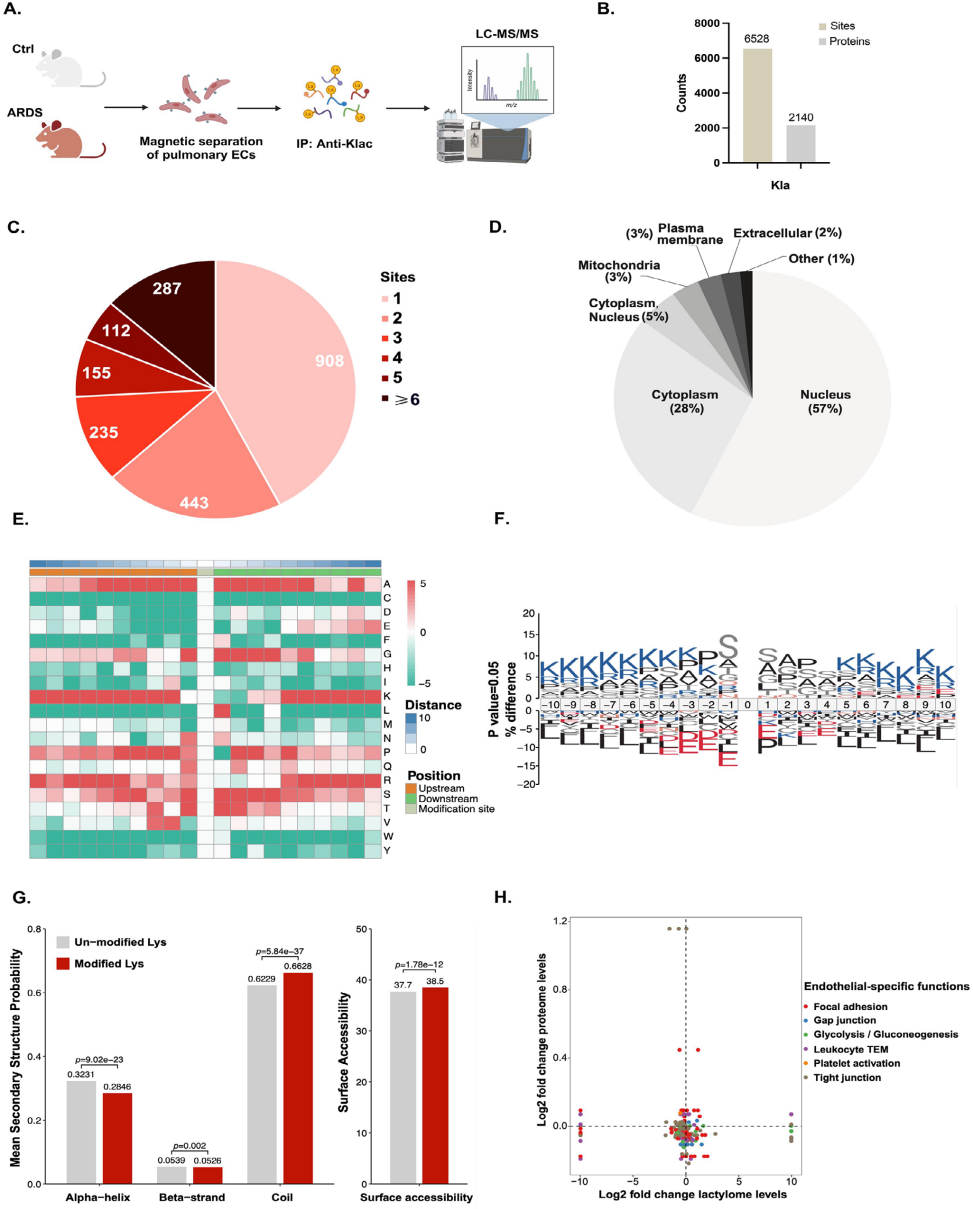
Functional landscape of the PEC lactylome. (A) Schematic representation of the experimental design (created using Biorender.com). (B) Lactylation sites and proteins identified in PECs. (C) Distribution of identified Klac sites per protein. (D) Subcellular locations of identified lactylated proteins in PECs. (E) Motif analysis of all identified Klac proteins. (F) Icelogo representation of flanking sequence preferences for all Klac sites. (G) Distribution of unlactylated and lactylated sites in protein‐structured regions. (H) Differential expression of Klac sites on proteins that participate in endothelium‐specific functions.

To further examine the role of Klac in PECs, we identified Klac substrates in PECs from control and ARDS mice (Figure 4A). A total of 6528 Klac sites in 2140 proteins were detected (Figure 4B). Among these proteins, 42% (908/2140) had a single modified site, while 13% (287/2140) exhibited more than six lactylation sites (Figure 4C). Subcellular localization analysis revealed that 57% (1220/2140) of the lactylated proteins were distributed in the nucleus, 28% (599/2140) in the cytoplasm, and 5% (107/2140) in both compartments (Figure 4D).

Given the specific region of enzyme‐substrate binding, we next analyzed the key amino acids flanking the identified Klac sites using iceLogo and Motif‐X algorithms (Figure 4E). The most overrepresented motif was SKxxxxxxK (Figure 4F and Figure S8). Using NetSurfP, we further assessed the structural characteristics of the lactylation sites. Approximately 66% of these sites were located in coils, 5% in strands, and 28% in helices, with lactylated residues showing a preference for coil regions compared with unlactylated residues. In addition, the average surface accessibility of Klac sites was significantly higher than that of unlactylated lysine residues (Figure 4G). Functional enrichment analysis revealed that Klac interfered with proteins involved in endothelium‐specific processes, including focal adhesion, gap junctions, glycolysis, leukocyte transendothelial migration, platelet activation, and tight junctions (Figure 4H).

Overall, these data indicate that Klac plays a significant role in PEC biology and may regulate critical endothelium‐specific functions.

### 2.5 Eno1 is Hyperlactylated at K193 in Dysfunctional PECs

**FIGURE 5:**
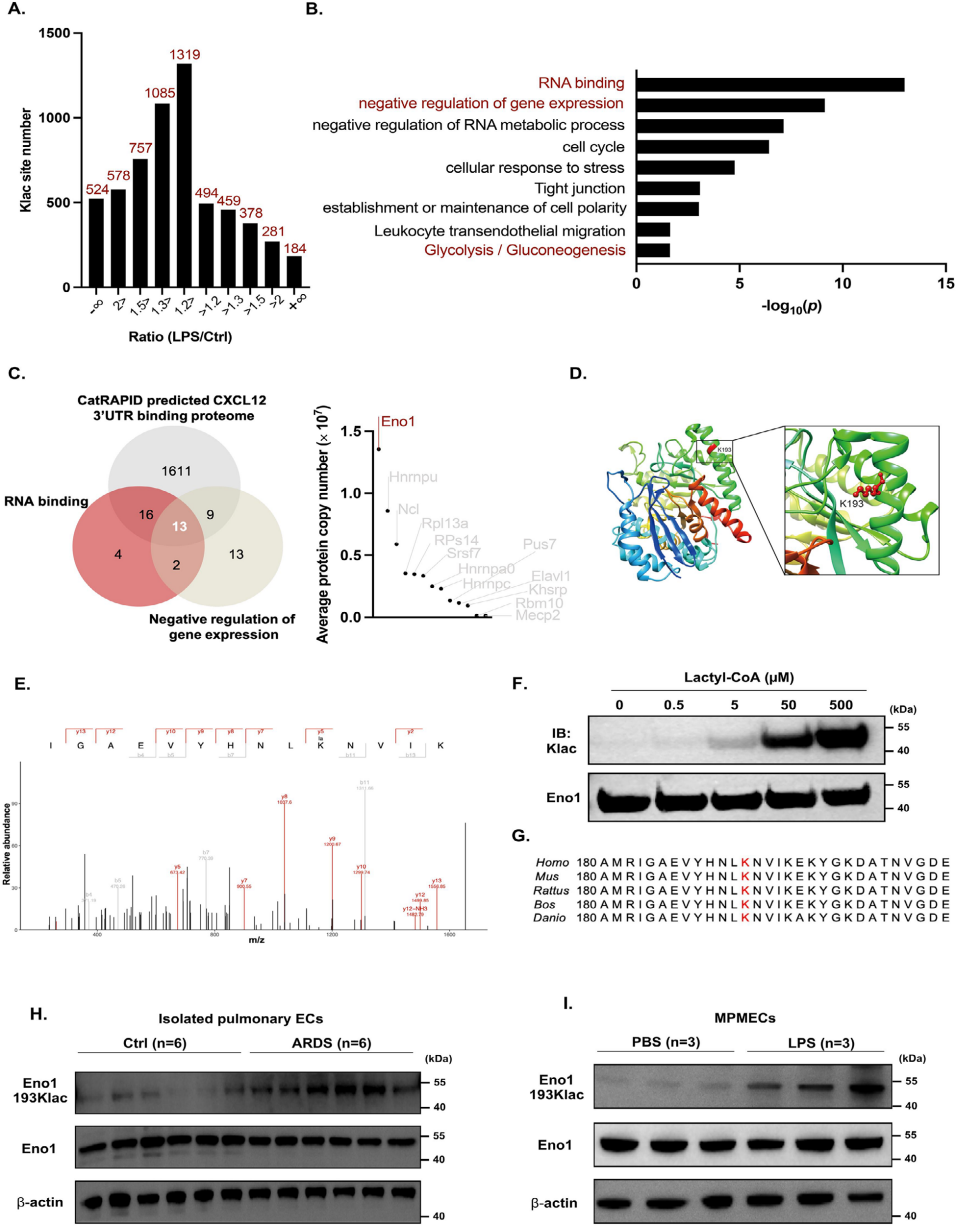
Increased protein lactylation in the PECs of ARDS mice, with Eno1 as a substrate. (A) Histogram of the ratio distribution of quantifiable Klac sites in the PECs of control and LPS‐induced ARDS mice. (B) GO items associated with Klac sites (ARDS/Control > +∞) located proteins. (C) Venn diagram of predicted CXCL12 3′ UTR binding proteins and proteins in picked GO items and the average protein copies of 13 screened proteins in PECs. (D) Ribbon diagram of the crystal structure of mouse Eno1 and lactylation at the K193 residue. (E) Mass spectrometry of Eno1 lactylated at K193. (F) Purified mouse Eno1 (100 µg/mL) was incubated with different concentrations of lactyl‐CoA for 1 h at 37°C. Then, the mixtures were added to protein loading buffer for denaturation, and K‐lactylation was assessed by western blotting. (G) The K193 site in Eno1 is conserved in different species. Conserved K193 sites are marked in red in the sequences among different species. (H) Western blotting analysis of the K193 lactylated level in PECs isolated from the lungs of control and LPS‐induced ARDS mice. (I) In vitro, the K193 lactylation levels in PBS‐ and LPS‐activated MPMECs were measured by western blotting.

Next, we quantified changes in Klac levels relative to total protein abundance in PECs. The analysis revealed that a total of 281 Klac sites in 231 proteins were significantly upregulated in the PECs of ARDS mice (cut‐off ratio > 2), while 578 Klac sites in 459 proteins were downregulated (cut‐off ratio < 0.5). Notably, glycolysis inhibition suppressed ARDS progression by reducing global lactylation levels in the PECs. This result suggested that upregulated Klac proteins contribute to PEC dysfunction. Intriguingly, 184 upregulated Klac sites, distributed on 169 proteins, were exclusively detected in the PECs of ARDS mice (Figure 5A). Gene Ontology (GO) analysis of these 169 proteins indicated their involvement in RNA binding, negative regulation of gene expression, cell cycle regulation, and glycolysis (Figure 5B). Further functional enrichment analysis indicated that these 169 proteins were involved in several endothelial‐associated biological processes, including tight junctions, leukocyte trans‐endothelial migration, endothelial cell (EC) migration, and establishment of the endothelial barrier (Figure S9).

Previous studies have shown that RNA‐binding proteins (RBPs) can modulate mRNA translation by interacting with the 3′ untranslated region (3′ UTR). To identify candidates, we combined the predicted CXCL12 3′ UTR‐binding proteome (via CatRAPID) with lactylation‐upregulated proteins enriched in relevant GO items. This analysis identified 13 lactylated proteins potentially capable of binding to the CXCL12 3′ UTR to regulate its translation. Among these, Eno1 exhibited significantly higher expression levels than the other candidates (Figure 5C). Eno1, a key glycolytic enzyme, has been shown to directly bind to target mRNAs to regulate their degradation or translation. Given the crucial role of glycolysis in endothelial dysfunction and Eno1's RNA‐binding capability, we selected Eno1 for further investigation to explore the role of lactylation in CXCL12 production.

A ribbon diagram of the predicted crystal structure model of mouse K193‐lactylated Eno1, along with the MS/MS spectra of the lactylated peptide, is shown in Figure 5D,E. In vitro experiments demonstrated that Eno1 could be lactylated by lactyl‐CoA in a concentration‐dependent manner (Figure 5F). In addition, the K193 residue is highly conserved across various species (Figure 5G).

To directly measure the lactylation level at the K193 residue of Eno1, we developed a specialized polyclonal antibody. Dot plot assays confirmed that this antibody specifically recognized the lactylated K193 peptide on Eno1 (Figure S10A). Further analysis revealed that K193 lactylation on Eno1 was significantly upregulated not only in the PECs of ARDS mice (Figure 5H and Figure S10B,C), but also in LPS‐treated MPMECs (Figure 5I) and HULEC‐5a cells (Figure S10D) in vitro.

### 2.6 K193 Lactylation Weakens Eno1 Binding to CXCL12 mRNA

**FIGURE 6:**
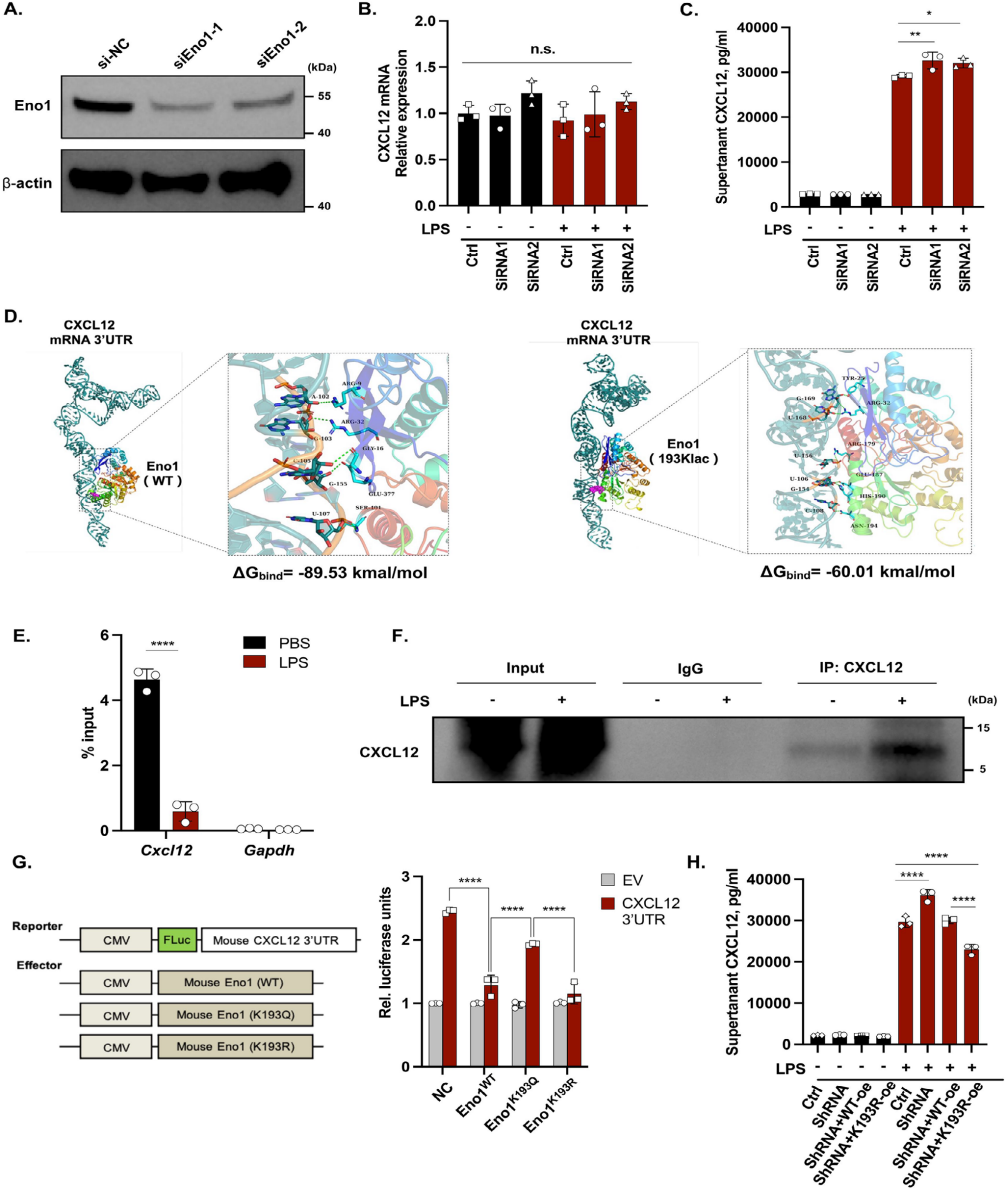
K193 lactylated Eno1 released the bound CXCL12 mRNA to accelerate translation. (A) Eno1 expression in MPMECs transfected with siRNAs. (B) CXCL12 mRNA and (C) protein expression in Eno1 KD cells after LPS stimulation (*n* = 3). (D) Molecular docking and ΔG binding energy calculation (E) RIP‐qPCR detection of Eno1‐bound CXCL12 transcripts in MPMECs treated with LPS (*n* = 3). (F) Click‐iT AHA assays to detect the CXCL12 translation efficacy in MPMECs treated with LPS (*n* = 3). (G) Schematics of the reporter and effector constructs. Dual‐luciferase analysis in HEK293T cells transfected with the reporter expressing the CXCL12‐3′ UTR and constructs expressing the WT, K193Q, or K193R Eno1 (*n* = 3). (H) CXCL12 concentrations in culture media from MPMECs transfected with shEno1, shEno1+oe‐WT, or shEno1+oe‐K193R (*n* = 3). *p < 0.05, **p < 0.01, ****p < 0.001.

To explore whether Eno1 directly regulates CXCL12 expression at the post‐transcriptional level, we used two independent small interfering RNAs (siRNAs) to knock down Eno1 expression in MPMECs. Both siRNAs effectively reduced Eno1 protein levels (Figure 6A). Although CXCL12 transcript levels remained unchanged (Figure 6B), a significant increase in CXCL12 protein expression was observed following LPS treatment (Figure 6C). These findings suggest that Eno1 suppresses CXCL12 expression via its RNA‐binding activity.

Next, we investigated the impact of K193 lactylation on the RNA binding capacity of Eno1. Molecular modeling and molecular dynamics simulations revealed that K193 lactylation altered the secondary structures of Eno1 (Figure S11A, B), reducing its compactness and increasing its flexibility (Figure S11C–F). Protein‐RNA docking simulations indicated that K193 lactylation weakened the interaction between Eno1 and the CXCL12 3′ UTR (Figure 6D), a finding corroborated by RNA immunoprecipitation experiments (Figure 6E). In addition, the Click‐iT (L‐azidohomoalanine) assay revealed enhanced CXCL12 translation after its release from Eno1 (Figure 6F). Dual‐reporter assays further supported this observation. To mimic Eno1 K193 lactylation, we generated a K193 glutamine (K193Q) mutant. We also generated a K193 arginine (K193R) mutant that was unable to undergo lactylation (Figure 6G). The results demonstrated that luciferase reporters fused to the CXCL12 3′ UTR were significantly suppressed by wild‐type (WT) Eno1 and K193R Eno1, but not K193Q Eno1.

We further knocked down Eno1 expression in MPMECs using lentiviral transduction and subsequently overexpressed either WT Eno1 or K193R Eno1 using adenoviruses (Figure S12A,B). Following LPS stimulation, the lactylation levels of K193R Eno1 significantly decreased compared with those of WT Eno1 (Figure S12C). Moreover, overexpression (oe) of K193R Eno1 in MPMECs resulted in lower CXCL12 expression compared with that observed in the WT Eno1‐oe group (Figure 6H).

Taken together, these findings demonstrate that Eno1 suppresses CXCL12 translation as an RBP, and this ability is impaired by K193 lactylation.

### 2.7 K193 Lactylation on Eno1 Increases the Enzyme's Activity

**FIGURE 7:**
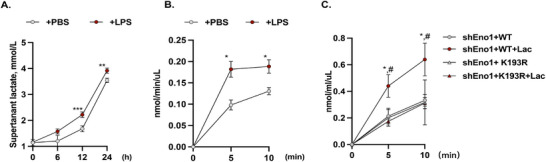
K193 lactylation augmented Eno1 enzymatic activity. (A) The levels of lactate in the culture medium of PBS‐ and LPS‐treated MPMECs over time (*n* = 3). (B) PBS‐ and LPS‐treated MPMECs lysates were prepared to measure Eno1 enzymatic activity by monitoring phosphoenolpyruvate concentrations over time (*n* = 3). (C) Eno1 enzymatic activity was measured in lysates from shEno1+oe‐WT and shEno1+oe‐K193R MPMEC (*n* = 6). *p < 0.05. In C, *compared with the untreated shEno1+oe‐WT group, *p < 0.05; ^#^compared with the lactate‐treated shEno1+oe‐K193R group, ^#^ *p *< 0.05.

We next investigated the role of K193 lactylation in regulating Eno1's enzymatic function. LPS treatment enhanced the glycolytic flux in MPMECs (Figure 7A), and while Eno1 expression remained unchanged (Figure 5I), its enzymatic activity significantly increased (Figure 7B). We hypothesized that K193 lactylation contributes to increased enzymatic activity.

To test this, we treated WT Eno1‐oe and K193R Eno1‐oe MPMECs with lactate directly. In WT Eno1‐oe MPMECs, lactate treatment significantly enhanced Eno1 enzymatic activity. By contrast, lactate had no effect on the enzymatic activity of Eno1 in K193R Eno1‐oe MPMECs (Figure 7C).

Overall, these results indicate that K193 lactylation enhances Eno1 enzymatic activity and promotes its dissociation from the CXCL12 3′ UTR.

## 3 Discussion

**FIGURE 8:**
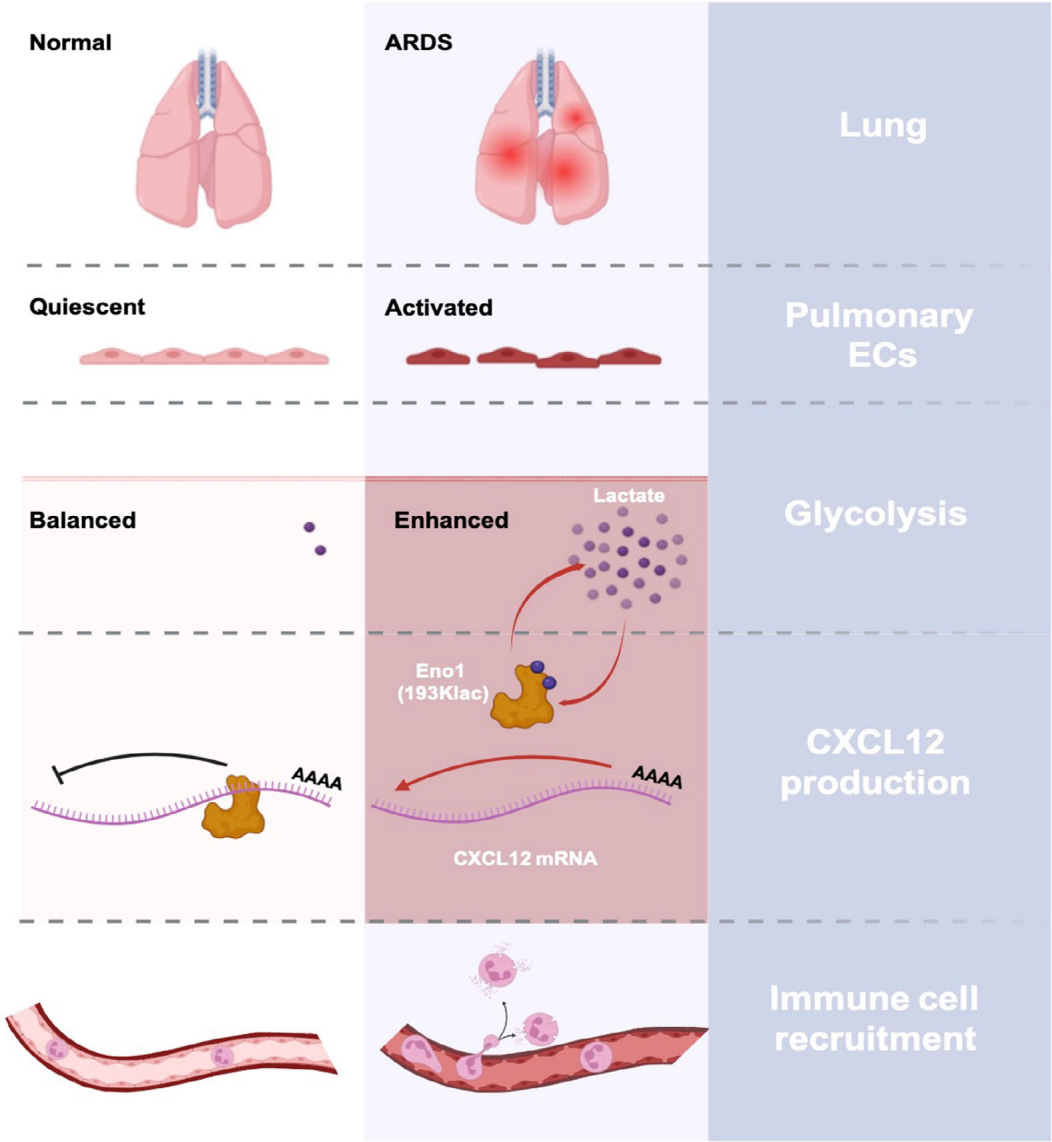
Scenario of this study (created using Biorender.com).

PECs play a crucial role in the development of ARDS. Pathogenetic factors and cytotoxic molecules in the pulmonary microenvironment directly stimulate PECs, triggering a cascade of events in the progression of ARDS [18, 19, 20]. In this present work, we demonstrated that lactylation, a novel PTM derived from lactate, plays an undiscovered role in aggravating PEC dysfunction and amplifying lung injuries during ARDS. The scenario is presented in Figure 8. Considering the metabolic reprogramming in the pulmonary microenvironment and lactate accumulation, we believe the role of Klac in ARDS progression and patient outcome has been largely neglected.

It is well established that ECs act as the gatekeepers of tissue lactate metabolism [21, 22, 23]. ECs convert most glucose to lactate because glycolysis is their main energy‐producing mechanism, regardless of oxygen concentration [24, 25]. Glycolysis can help ECs use less oxygen, facilitating its transfer to perivascular cells. Moreover, glycolysis generates fewer reactive oxygen species, which may induce cell apoptosis [26]. In addition, glycolysis produces ATP more quickly than oxidative phosphorylation, which is essential for EC proliferation and angiogenesis [27]. Dysfunctional ECs enhance glycolysis in response to external stimuli, resulting in elevated lactate production. Lactate has long been recognized as a waste product and a sign of glycolysis. However, compelling evidence has shed light on the complexity of lactate as a functional molecule that manipulates cell responses under physiological and pathological conditions. We previously demonstrated that lactate upregulates ICAM‐1 expression in PECs to enhance leukocyte transendothelial migration in the lungs of the ARDS mice. Intriguingly, blockade or genetic knockdown of lactate sensing and signaling receptor G protein‐coupled receptor 81 only partially mitigates ICAM‐1 hyperexpression [28], indicating that the lactate‐mediated mechanisms responsible for the triggering and persistence of endothelial dysfunction in ARDS need to be further explored. Recently, lactate has been shown to serve as a substrate and a key determinant of a novel PTM called lactylation. Several studies have revealed the role of lactylation in multiple endothelial‐associated diseases, including myocardial infarction [29], atherosclerosis [30], diabetic retinopathy [31], and sepsis‐associated lung injury [32, 33]. Based on these facts, we hypothesized that lactylation plays a crucial role in PECs during ARDS. We observed that increasing lactylation amplifies the production of chemokines in PECs, especially CXCL12. Furthermore, we performed lactylome analysis using the PECs from ARDS mice. The results provided clues to explore the effects of lactylation on PEC dysfunction in patients with ARDS.

Among the upregulated Klac proteins in the PECs of ARDS mice, we found that the glycolytic enzyme Eno1 has a direct promotional effect on CXCL12 production. Eno1 is the ninth rate‐limiting glycolytic enzyme and is reported to be associated with disturbed shear‐induced endothelial‐to‐mesenchymal transition [34] and chronic hypoxia‐induced endothelium dysfunction [35]. As a moonlighting protein, Eno1 plays multiple roles aside from its catalytic function [36]. Moreover, numerous studies have shed light on its oncogenic function as an RBP. Carpousis et al. found that Eno1 is a component of the RNA degradosome that mediates RNA metabolism in *Escherichia coli* [37]. Sun et al. [38] reported that Eno1 promotes yes‐associated protein 1 (YAP1) translation as an RBP to accelerate liver carcinogenesis. Zhang et al. [39] documented that Eno1 binds to iron‐regulated protein 1 (IRF1) mRNA as an RBP to promote its degradation, thereby suppressing cancer cell ferroptosis. Furthermore, a recent study by Wang et al. [40] showed that Eno1 interacts with various mRNAs as an RBP to affect gastric cancer cell proliferation, migration, and apoptosis. Nonetheless, the function of Eno1 as an RBP in PEC dysfunction has, to the best of our knowledge, not yet been investigated. In this work, the presented data supported that Eno1 binds to the CXCL12 3′ UTR to repress translation in resting PECs and that K193 lactylation on Eno1 decreases this affinity to promote translation following PEC activation. Lactylation‐induced changes enhance Eno1 glycolytic activity, helping ECs to adapt to inflammatory stimulation, while the released CXCL12 mRNAs are translated more efficiently in response to activation. However, considering the high expression levels of Eno1 in PECs, the role of its RNA‐binding in pulmonary physiology and pathology is worthy of further investigation.

PTMs have significant impacts on almost all aspects of protein structure and function [41]. Once the shape changes, the functions of proteins always change because their structure determines their interactivity with other molecules. Over the past decades, more than 10 types of PTMs on Eno1 have been reported in different cells. Huppertz et al. [42] reported that the acetylation of Eno1 at K89, K92, and K105 residues changes its conformation and then enhances its RNA binding capacity. Lactylation is a novel PTM, and its impact on protein structure has not yet been explored. In our study, we performed molecular docking and dynamics analyses to investigate the influence of K193 lactylation on Eno1. K193 lactylation drives Eno1 to a more “loosened state,” which decreases its affinity to the bound CXCL12 3′ UTR, thus promoting translation. The 3′ UTR is the 3′ noncoding region of mRNA and controls mRNA translation, localization, degradation, and even protein–protein interactions [43]. Accumulating evidence emphasizes the importance of 3′ UTR‐mediated translation control in diseases. The 3′ UTR controls translation, requiring the binding of RBPs. Previous studies have verified various mechanisms by which RBPs can bind to the 3′ UTRs of mRNAs to inhibit translation. Further investigations should be conducted to explore whether and how Eno1 silences CXCL12 translation in resting PECs.

CXCL12 is a member of the CXC chemokine family and is constitutively expressed at low levels by various types of ECs, including PECs. Chandrasekaran et al. [44] demonstrated the critical role of CXCL12 in pulmonary vascular development, with the absence of CXCL12 causing distal vascular hypoplasia. Moreover, there is growing evidence that CXCL12 exerts beneficial or harmful effects in a tissue‐ or context‐specific fashion. Wang et al. [45] reported that brain endothelial‐derived CXCL12 can attract protective natural killer cells to improve behavioral deficits in ischemic stroke mouse models. However, Döring et al. [46] reported that arterial endothelial‐derived CXCL12 is responsible for atherosclerosis in coronary artery disease. Recent studies have shown that CXCL12 is a potent chemokine for selective marrow neutrophil recruitment [47] into the lungs to sustain inflammation in ARDS, and blockade of CXCL12 alleviates lung injury and improves the survival rates of ARDS mice [48, 49]. At present, the mechanisms by which pathogenic stimuli regulate CXCL12 expression in PECs during ARDS remain largely unknown. Our data provide mechanistic insight into the PTM‐regulated expression of CXCL12 in PECs.

In summary, this work revealed a previously unknown pathogenetic role of lactylation in manipulating PEC activation and ARDS progression. We characterized the lactylome of PECs and explored the function of K193 lactylation of Eno1 in the activated endothelium. Our results also indicated that Eno1 (K193) is a potential therapeutic target in ARDS, but its clinical application value needs to be further validated.

### 3.1 Limitations of This Study

We systematically explored the role of lactylation in PEC CXCL12 production in ARDS. However, this study has some limitations. First, the sample size of patients was small, which may limit the generalizability of the conclusions. Second, no single animal model can replicate the complexities of clinical patients with ARDS. Thus, these findings should be further confirmed using more types of ARDS models, such as the oleic acid‐induced ARDS model and the two‐hit ARDS model. Third, mouse models with PEC‐targeted Eno1 K193R mutations are required to validate these findings definitively. Finally, we focused only on the role of lactylation in CXCL12 production in activated PECs. However, the effects of lactylation on other endothelial‐associated biological processes (e.g., endothelial barrier formation) require further exploration.

## 4 Materials and Methods

### 4.1 Patient Enrollment

This retrospective cohort study was conducted from September 2021 to January 2023. Patients who met the following criteria were included in this study: (1) adult patients aged > 18 years; (2) patient diagnosis of ARDS according to the Berlin criteria within 72 h of admission; and (3) patients in whom an artificial airway was established, including intubation and tracheostomy. The exclusion criteria were as follows: (1) chronic pulmonary disease, including chronic obstructive pulmonary disease (COPD), asthma, and pulmonary fibrosis; (2) pregnancy, malignancy, and autoimmune diseases; (3) contraindications to fibrobronchoscope examinations; and (4) an expected survival of less than 24 h. A cohort of patients who underwent uvulopalatopharyngoplasty with intubation and mechanical ventilation but without previous pulmonary diseases was enrolled as controls.

### 4.2 Human Samples

BALF was collected on the first day of intensive care unit (ICU) admission. Briefly, 40 mL of normal saline was lavaged in the mid lobe on the right and the upper lobes on the left, and the fluid was then collected, with recovery of more than 40% of the original volume. Serum lactate levels were obtained simultaneously and sent to the hospital's central laboratory. Arterial blood gas analyses were performed by bedside point‐of‐care analysis and obtained whenever necessary. BALF samples were centrifuged (1000 × rpm, 5 min, 4°C), and supernatants were stored at −80°C for batch analysis.

### 4.3 Animals

C57/BL6 mice (6–8 weeks of age) were purchased from GemPharmatech (China) and bred in the Division of Laboratory Animal Center at Southeast University (Nanjing, Jiangsu, China).

### 4.4 Experimental ARDS Model and Treatments

LPS‐ or HCl‐induced ARDS has been described previously. Briefly, following anesthesia with pentobarbital, LPS (dissolved in sterilized phosphate‐buffered saline [PBS], 5 mg/kg body weight, O55:B5, Millipore Sigma, USA) or HCL (1.5 mL/kg body weight, 0.1 N, pH 1.4) was delivered into the lung via tracheostomy, and the incision was closed with 4‐0 silk. After the procedures, mice were rewarmed until fully awake and were then returned to their cages. CLP‐induced ARDS was performed as described elsewhere with minor modifications. Briefly, following continuous anesthesia with isoflurane, a 1 cm midline abdominal incision was performed, and the cecum was ligated and punctured twice with a 21‐gauge needle. After the procedures, the cecum was returned to the peritoneal cavity, and the incision was closed with 4‐0 silk.

Lactate (pH 6.8, 0.5 g/kg body weight, HY‐B2227, MedChemExpress, USA) was injected intraperitoneally 6 h after procedures in the mouse model. OXA (0.5 g/kg body weight, HY‐W013032A, MedChemExpress, USA) was injected intraperitoneally 6 h before procedures to inhibit lactylation. BALF was collected via tracheal intubation. BALF samples were prepared and stored as described above. Half of the right lung was rinsed with PBS and homogenized in 10 mL PBS using an ultrasonic mill. The homogenate was centrifuged (5000 × *g*, 5 min, 4°C) and the supernatant was stored at −80°C for further analysis. For survival analysis, mice were intratracheally injected with a higher dose (25 mg/kg) of LPS.

### 4.5 Colorimetric Assay

Lactate and NO levels in samples were determined by colorimetric assays following the manufacturers’ instructions (Lactic acid assay kit, A019‐2‐1, Nanjing Jiancheng Bioengineering Institute, China; NO assay kit, S0021S, Beyotime, China). Total proteins in the BALF and homogenate were determined using the bicinchoninic acid (BCA) assay to normalize the substance levels.

### 4.6 Quantitative Real‐Time PCR

Total mRNA was isolated from lung tissue or cultured cells using a SPARKeasy kit (AC0201, SparkJade, China) in accordance with the manufacturer's instructions. The mRNA was converted to cDNA using HiScript III RT SuperMix (R323, Vazyme, China) and then processed for real‐time polymerase chain reaction with ChamQ SYBR qPCR Master Mix (Q341‐02, Vazyme, China). The ΔΔ*C*_t_ method was used to analyze the data, and the relative expression levels of all genes were normalized to β‐actin. The primers used in this study were as follows:

Mouse‐*Il1β*
F: GCAACTGTTCCTGAACTCAACT
R: ATCTTTTGGGGTCCGTCAACT
Mouse‐*Il6*
F: TAGTCCTTCCTACCCCAATTTCC
R: TTGGTCCTTAGCCACTCCTTC
Mouse‐*Cxcl12*
F: TCGAGAAAGACGGGGAAGTAA
R: ACCACACACACCCCACTAACA
Mouse‐*β‐actin*
F: GGCTGTATTCCCCTCCATCG
R: CCAGTTGGTAACAATGCCATGT
Human‐*Cxcl12*
F: ATTCTCAACACTCCAAACTGTGC
R: ACTTTAGCTTCGGGTCAATGC
Human‐*β‐actin*
F: CTCGCCTTTGCCGATCC
R: ATCCTTCTGACCCATGCCC

### 4.7 Enzyme‐Linked Immunosorbent Assay

ILs, chemokines, vWF, and CXCL12 levels in samples were determined by Enzyme‐Linked Immunosorbent Assay (ELISA) following the manufacturer's protocols (Elabscience, China; ABclonal, China) and were standardized according to the total protein in each sample.

### 4.8 H&E and Immunofluorescence Staining

Lung samples were first fixed in 4% paraformaldehyde for 48 h and then processed into paraffin. For H&E staining, sections (4 µm in thickness) were stained directly with dye. For immunofluorescence, sections were permeabilized in 0.5% Triton X‐100 for 5 min, and then blocked with 10% normal goat serum for 1 h at room temperature. After washing with PBS, the sections were incubated with primary antibodies at 4°C overnight. The primary antibodies were goat anti‐L‐lactyl lysine (PTM‐1401, PTM Biolabs, China), rabbit anti‐K193 lactylated Eno1 (custom designed, ABclonal, China), and rat anti‐mouse CD31 (ab222783, Abcam, USA). After extensive washing with PBS, the sections were incubated with fluorescently conjugated secondary antibodies at a 1:500 dilution for 1 h at room temperature and then washed before observation by confocal microscopy.

### 4.9 Flow Cytometry and Analysis

The cells in BALF were incubated with purified rat anti‐mouse CD16/CD32 for 10 min at room temperature and then washed once with PBS. Subsequently, the cells were stained with fixable viability dye, rat anti‐mouse CD45, rat anti‐mouse CD11b, and rat anti‐mouse Ly6G under the manufacturer's instructions. Eventually, the cells were processed using the LSRFortessa flow cytometer, and the generated data were analyzed by FlowJo software.

### 4.10 Primary Mouse PEC Isolation

Primary mouse PECs were isolated as previously described with some modifications. Briefly, peripheral lung tissues from control or ARDS mice were chopped into small pieces and then digested with collagenase I. Cell suspensions were filtered through 70‐µm strainers and then depleted of CD45‐positive cells using a commercial kit (18945, Stemcell, USA). The remaining CD45‐negative cells were then incubated with CD31 MicroBeads (130‐097‐418, Miltenyi, USA) to isolate ECs according to the manufacturer's instructions. The purity of isolated ECs was over 90%.

### 4.11 Western Blotting

Western blotting was performed as previously described elsewhere. The following primary antibodies were used: anti‐L‐lactyl lysine (PTM‐1401, PTM Biolabs, China), anti‐mouse Eno1 (A1033, ABclonal, China), anti‐K193 lactylated Eno1 (custom designed, ABclonal, China), and anti‐mouse β‐actin (ZF0033, ZFdows Bio, China). The signals were analyzed and quantified using a gel imaging system (Chemi4800mini, Bioshine, China).

### 4.12 BM Immune Cell Isolation

This experiment has been described elsewhere [50]. Briefly, the mouse femurs were flushed out with ice‐cold PBS onto a 40‐µm nylon cell strainer until the flow‐through turned transparent. After smashing and washing, the cells were centrifuged (1500 × rpm, 5 min, 4°C) and resuspended in 1 × red blood lysis buffer. Cells were ready for use after neutralization, washing, and counting.

### 4.13 Cell Culture

The MPMECs and HULEC‐5a cells (from ATCC) were cultured in an EC medium kit (1001, Sciencell, USA). BM immune cells were cultured in Roswell Park Memorial Institute (RPMI) 1640 (11875119, Gibco, USA) with 10% fetal bovine serum (FBS). HEK293T cells (from ATCC) were cultured in Dulbecco's Modified Eagle Medium (DMEM) (11965092, Gibco, USA) with 10% FBS (F8318, Sigma‐Aldrich, Australia).

### 4.14 Migration Assay

MPMECs were seeded in 24‐well plates and randomly divided into four groups: (1) control group, in which cells were treated with PBS; (2) LPS group, in which cells were treated with LPS (1 µg/mL) for 24 h; (3) LPS+Lac group, in which cells were pretreated with LPS for 6 h, before adding lactate for another 18 h; and (4) LPS+OXA, in which cells were pretreated with OXA for 6 h, before adding LPS for another 24 h. The MPMECs were washed with PBS and cultured in fresh EC medium. BM immune cells were seeded into the top chamber of an 8‐µm Transwell and cocultured with MPMECs for 8 h. The migrated BM cells in the bottom chamber were then counted.

### 4.15 Chemokine Beads Assay (Luminex)

Cell culture supernatants under different treatments were collected for a multi‐cytokine assay. The chemokine levels were evaluated using the Luminex 200 system (Luminex) in accordance with the manufacturer's recommendations.

### 4.16 Lactylation LC‐MS/MS Analysis

Cells were lysed with an ultrasonic processor and then digested with trypsin. An anti‐L‐lactyl lysine antibody was used to enrich Kla‐modified peptides. After drying and desalting, tryptic peptides were exposed to a capillary source and then processed for MS (timsTOF Pro, Bruker Daltonics, Germany). The MS/MS data were analyzed using the MaxQuant search engine (v.1.6.15.0). The quantified Kla peptide ratios were adjusted to their respective protein expression levels. The LC‐MS/MS and partial bioinformatics analyses were conducted blindly by PTM Biolabs (China).

The mass spectrometry proteomics data have been deposited in the ProteomeXchange Consortium via the iProX partner repository with the dataset identifier PXD063694.

### 4.17 Molecular Dynamics Simulation and Docking

The structure of Eno1 (Uniprot ID: P17182) was predicted directly in AlphaFold2. Protonation treatment was conducted at pH 6.5 by the H++3 online server. Lactylation was performed at residue K193, where the N‐terminus of K193 was formed by an amide bond with the carboxyl group of L‐lactate. Molecular dynamics simulations were conducted using Gromacs 5.1.5. The simulation system was set in a closed environment with a temperature of 289.15 K (25°C), a pH of 6.5, and a pressure of 1 bar. Periodic boundary conditions were set with the system center as the reference point, and the minimum distance from the box edge was set to 10 Å. The receptor structure topology file was converted into a GROMACS‐recognizable file using the pdb2gmx tool, with the force field parameter AMBEff14SB used for protein processing and the modified peptide force field parameters derived from the GAFF2 force field, along with TIP3P water molecules to simulate the water environment. Following the initial system construction, the steepest descent method was used to minimize the system energy for all atoms. A constant number of particles, pressure, and temperature (NPT) equilibrium simulation was conducted for a duration of 1000 ps with atom position constraints, followed by a 100 ns production dynamic simulation under NPT conditions, with the system simulated every 2 fs. Covalent bond lengths were constrained using a linear constraint solver, and long‐range electrostatic interactions were treated using the Particle Mesh Ewald (PME) method. The temperature and pressure were kept constant using a V‐rescale thermostat and a Parrinello–Rahman barostat, with a cut‐off radius of 12 Å for neighbor searching and nonbonded interactions, and the LINCS algorithm was used for all bonds.

The 3D structure of the 3′ UTR mRNA of CXCL12 was constructed using RNAComposer based on the secondary structure generated by RNAfold. Molecular docking was performed using HDOCK and scored using ITSoreRP. A negative score indicates molecular binding, whereas a larger absolute value indicates stronger binding affinity. The maximum number of output configurations for docking was set to 100, and the top 10 configurations were scored. Confidence analysis was performed using a confidence score, where a score greater than 0.7 indicated reliable docking and a high likelihood of molecular binding. The top configuration with the best docking score and confidence scores was selected from the docking configurations for further analysis.

All simulation and docking results were visualized using the open‐source software PyMOL 2.04 with the help of PhadCalc (China).

### 4.18 Δ*G* Binding Energy Calculation

Molecular Mechanics with Generalized Born and Surface Area Solvation (MM/GBSA) was used to calculate the Δ*G* binding energy between proteins and RNA. The Δ*G* binding energy was calculated following the formulas:

(1) $$\Delta G_{\mathrm{bind}}=\Delta H-T\Delta S\approx \Delta G_{\mathrm{solv}}+\Delta G_{\mathrm{GAS}}-T\Delta S$$

(2) $$\Delta G_{\mathrm{GAS}}=\Delta E_{\mathrm{int}}+\Delta E_{\mathrm{vdw}}+\Delta E_{\mathrm{ele}}$$

(3) $$\Delta G_{\mathrm{solv}}=\Delta E_{\mathrm{surf}}+\Delta E_{\mathrm{GB}}$$

where Δ*G*_GAS_ represents the change in vacuum kinetic energy upon binding the receptor and ligand, and it is further subdivided into Δ*E*_int_, Δ*E*_vdw_, and Δ*E*_ele_. Δ*E*_int_ corresponds to alterations in bond, angle, and dihedral angle energy; Δ*E*_vdw_ indicates variations in van der Waals energy pre‐ and post‐binding; and Δ*E*_ele_ signifies shifts in electrostatic interaction. Δ*G*_solv_ consists of Δ*E*_surf_ and Δ*E*_GB_, representing the solvent effect. Δ*E*_surf_ is determined by calculating the solvent‐accessible surface area, while Δ*E*_GB_ is calculated by the APBS program. *T*Δ*S* was not considered when calculating the Δ*G*_bind_ because its contribution to binding energy was minimal.

### 4.19 RNA Immunoprecipitation

MPMECs (∼10^7^ cells) were treated with PBS or LPS (1 µg/mL) for 24 h. Cells were washed three times with ice‐cold PBS and then prepared using an RNA Immunoprecipitation (RIP) kit (Bes5101, BersinBio, China) according to the manufacturer's instructions. The RNA was then extracted via phenol–chloroform and then stored at −80°C.

### 4.20 siRNAs Transfection and Adenovirus Infection

The siRNAs to knock down Eno1 expression were designed and synthesized by RiboBio, Co. Ltd. (China), with a scramble siRNA used as a negative control. The siRNAs were transfected into MPMECs at a concentration of 30 nM with riboFECT CP (C10502‐05, RiboBio, China). After 48 h, the cells were processed for further experiments.

To overexpress Eno1 WT or K193R, adenoviruses subcloned with cDNA expressing WT or K193R Eno1 and sh‐Eno1 were constructed by Genepharma, Co. Ltd. (China).

### 4.21 Dual‐Luciferase Reporter Assay

The 3′ UTR of CXCL12 was amplified and inserted into the pGL3 dual‐luciferase reporter vector. HEK293T cells were seeded in 48‐well plates overnight before transfection. Either the Eno1 coding DNA sequence (CDS), the Eno1 K193R CDS, or the Eno1 K193Q CDS, along with the CXCL12 3′ UTR, was co‐transfected into cells using an advanced DNA/RNA transfection reagent (AD600100, Zeta‐life, USA). The luciferase activities of the different groups were measured 48 h later using a dual‐luciferase reporter assay system (E2940, Promega, USA). Renilla luciferase activity was used to normalize the firefly luciferase activity. All plasmids were provided by Genomeditech, Co. Ltd. (China).

### 4.22 Translation Assay

Click‐iT AHA (L‐azidohomoalanine) analysis was performed to detect newly synthesized proteins, as described previously [51], with slight modification. Briefly, MPMECs were pretreated with PBS or LPS (1 µg/mL). After 24 h, the cells were washed three times with PBS and then cultured in DMEM without methionine/cysteine (21013024, Gibco, USA) for 1 h, followed by culturing in DMEM with 5% FBS and 100 µg/mL AHA (C10102, Invitrogen, USA) for another 1 h. Subsequently, the cells were then harvested, lysed on ice for 30 min, and then centrifuged (16,000 × *g*, 15 min, 4°C). The supernatants were incubated with rabbit IgG or CXCL12 (ab25117, Abcam, USA) at 4°C overnight and then processed to incubate with A/G‐conjugated beads for 2 h. The beads were washed several times with lysis buffer and then incubated with Biotin‐PEG4‐alkyne (40 µM, 1458576‐00‐5, MedChemExpress, USA) in Click‐iT protein reaction buffer (C10276, Invitrogen, USA) following the manufacturer's instructions. The proteins were then extracted using methanol/chloroform and then analyzed by western blotting using streptavidin‐conjugated horseradish peroxidase (SA00001‐0, Proteintech, China).

### 4.23 Eno1 Activity Assay

The Eno1 activity assay kit (ab241024, Abcam, USA) was used to measure the activity of Eno1 in different processed MPMECs according to the manufacturer's instructions.

### 4.24 Statistical Analyses

Data are presented as the mean ± SD, mean ± SEM, or median (IQR) and were compared using the Student's *t*‐test or Mann–Whitney *U* test between two groups, or one‐way analysis of variance (ANOVA) or Kruskal–Wallis *H* test between three or more groups according to the data distributions. Categorical variables are presented as proportions or frequencies, as appropriate, and were compared using the chi‐square or Fisher's exact test, as appropriate. Survival to the end of the follow‐up period is expressed with Kaplan–Meier curves using the log‐rank test for between‐group analysis, with the cut‐off value determined by ROC curves, which were constructed to compare indicators in the prediction of mortality. J‐statistics were used to determine the cut‐off values by calculating the maximum of the Youden index (sensitivity + specificity − 1). Linear correlations between two parameters were analyzed using Pearson's or Spearman's tests, depending on the data distributions. A *p* < 0.05 level was considered to indicate statistical significance. The statistical parameters are provided in the figures and figure legends. All analyses were performed using GraphPad software (Prism v.8.0) or the statistical package IBM SPSS Statistics (version 24).

## Author Contributions

X.L., W.C., and W.N. planned this project and designed experiments. X.L., W.C. and X.D. carried out the human sample collection and tests. X.L. carried out animal experiments and most molecular experiments with the help of H.W. in ELISA experiments. X.L. carried out and analyzed the proteomic experiments. X.L. and W.C. prepared this manuscript. M.Z. and W.C. critically reviewed and revised this manuscript. All authors have read and approved the final manuscript.

## Ethics Statement

The samples of patients and related study were approved by the Research Ethics Board of Zhongda Hospital (Southeast University, Nanjing, China, 2021ZDSYLL215‐P01). Written informed consent was obtained from the next of kin of the participants. Besides, all animal procedures were approved by the Institutional Animal Care and Use Committee of Southeast University (No. 20220227005).

## Conflicts of Interest

The authors declare no conflicts of interest.

## Supporting information




**Supporting File**: mco270344‐sup‐0001‐SuppMat.docx

## Data Availability

All data needed to evaluate the conclusion in this paper are presented in the paper and/or in the Supporting Information. The data were available upon request from the corresponding authors.
