# Supplementary material for: Global Lactylome Reveals Lactylation‐Dependent Mechanisms Underlying CXC Motif Chemokine Ligand 12 Expression in Pulmonary Endothelium During Acute Respiratory Distress Syndrome
Source: MedComm (2020). 2025 Aug 29;6(9):e70344. doi: 10.1002/mco2.70344 (PMC12394890; doi:10.1002/mco2.70344)
Supplement: Supplementary file 1 — Supporting File: mco270344‐sup‐0001‐SuppMat.docx [file MCO2-6-e70344-s001.docx]

**
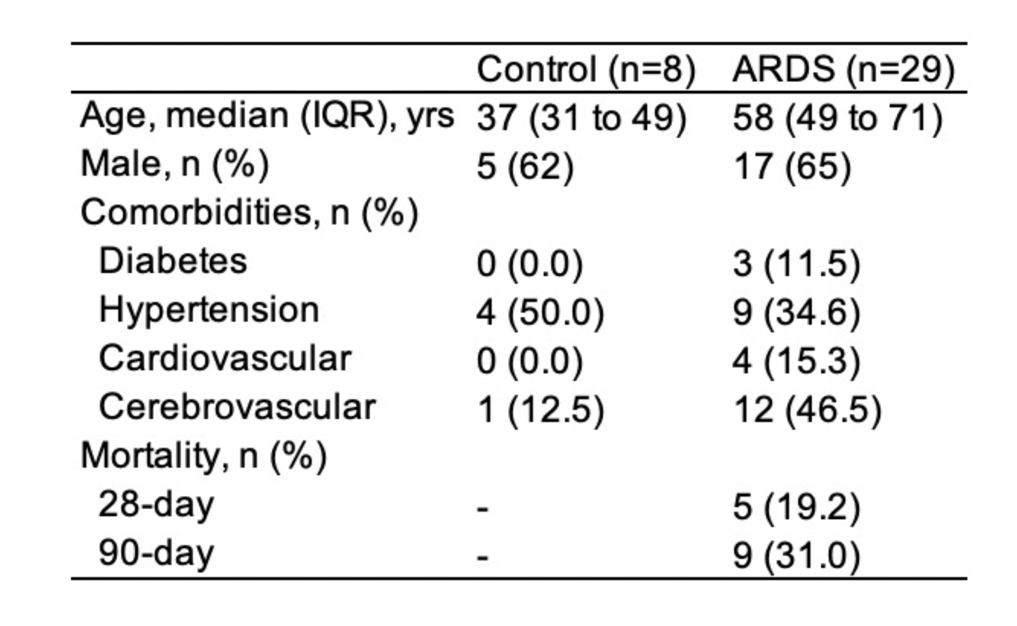
**

**Table S1 Characteristics of non-ARDS and ARDS patients.**

**
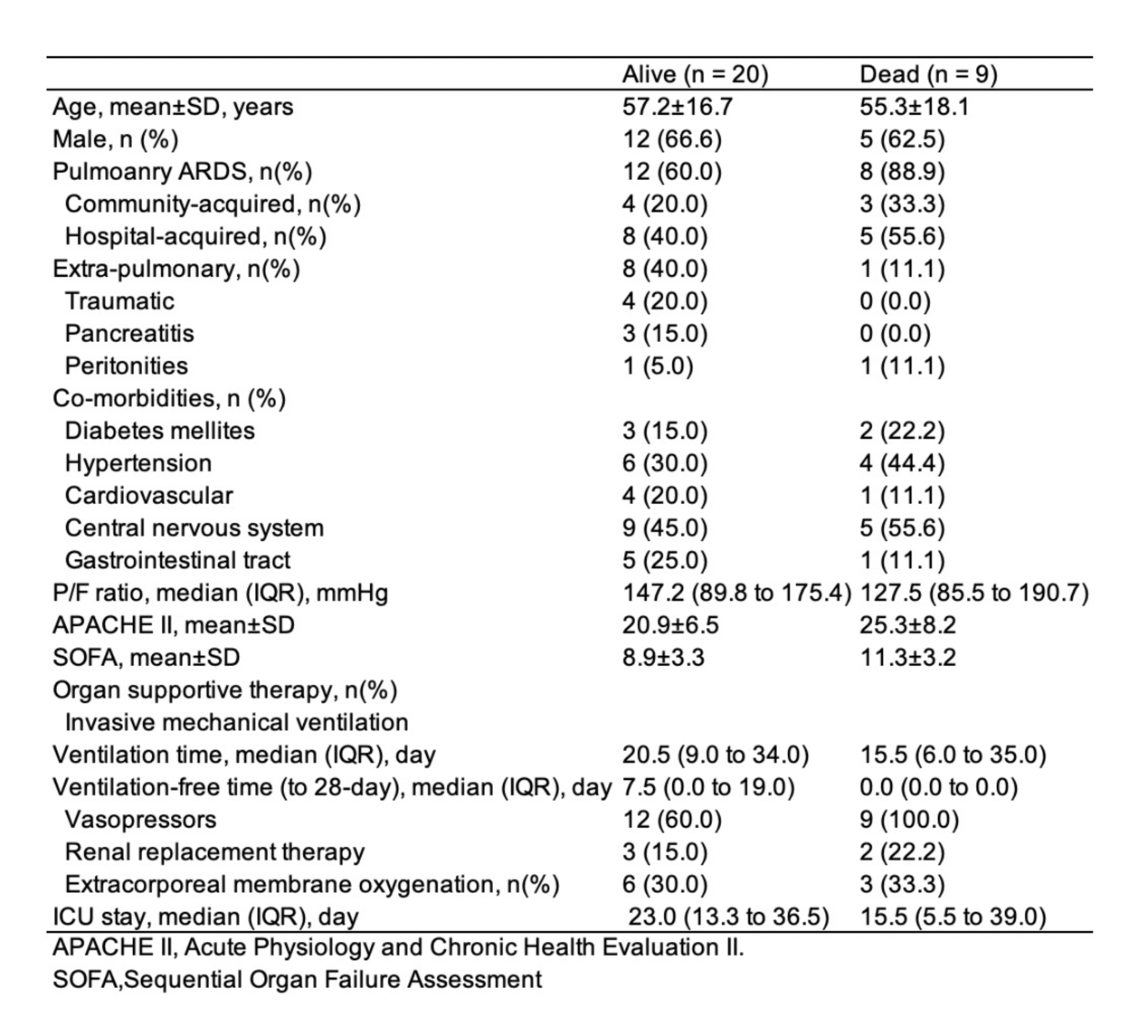
**

**Table S2 Characteristics of ARDS subgroups.**

**
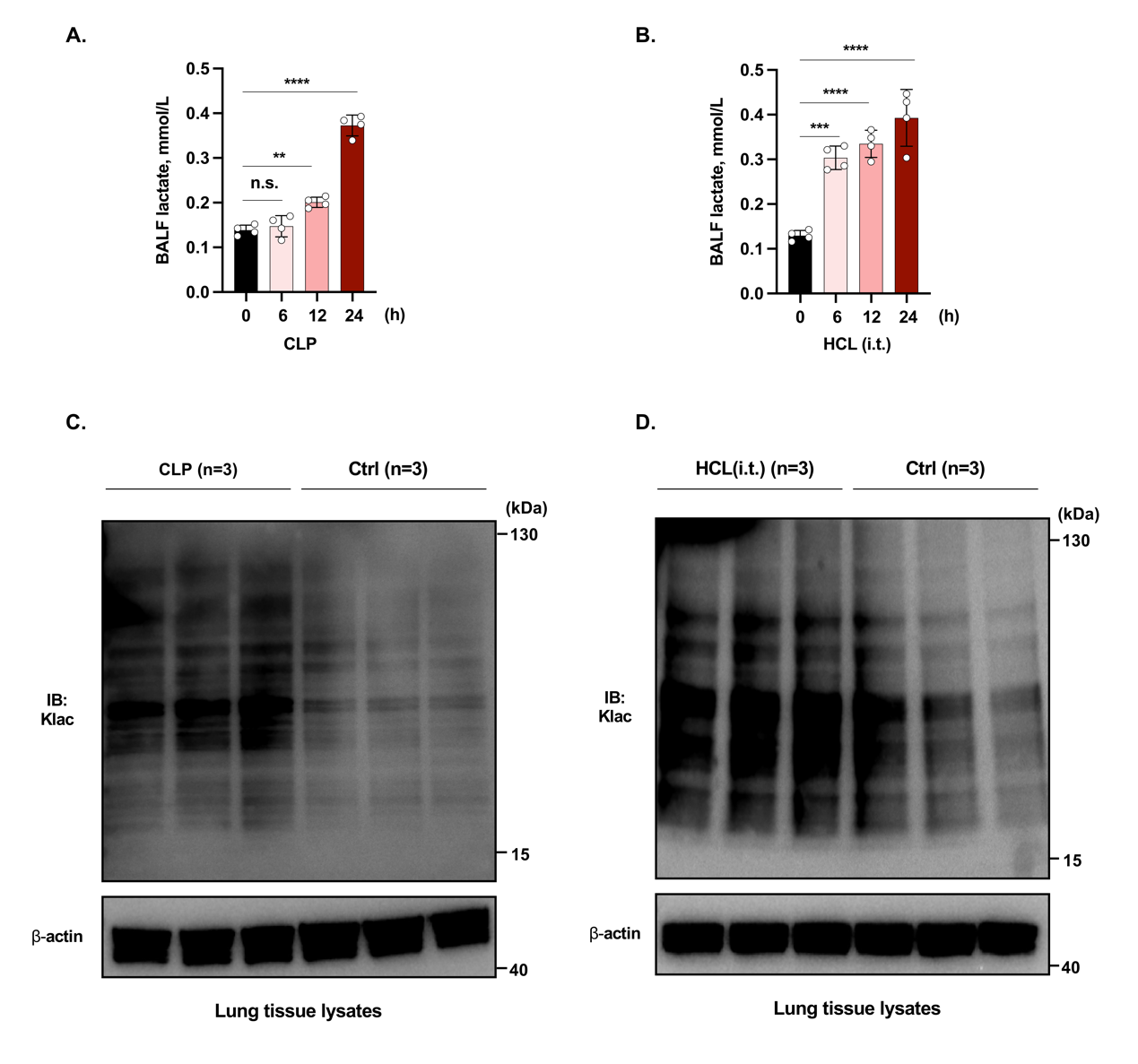
**

**Figure S1 Increased pulmonary lactylation levels in CLP-induced ARDS model and HCL-induced ARDS model.** (A) Lactate levels in the BALF samples of CLP-induced ARDS mice increased over time (n=4 per time point). (B) Lactate levels in the BALF samples of HCL-induced ARDS mice increased over time (n=4 per time point). (C) Western blotting of pan-Klac in lung tissue lysates from control mice and CLP-induced ARDS. (D) Western blotting of pan-Klac in lung tissue lysates from control mice and HCL-induced ARDS. ***p*<0.01; ****p*<0.005; *****p*<0.001.


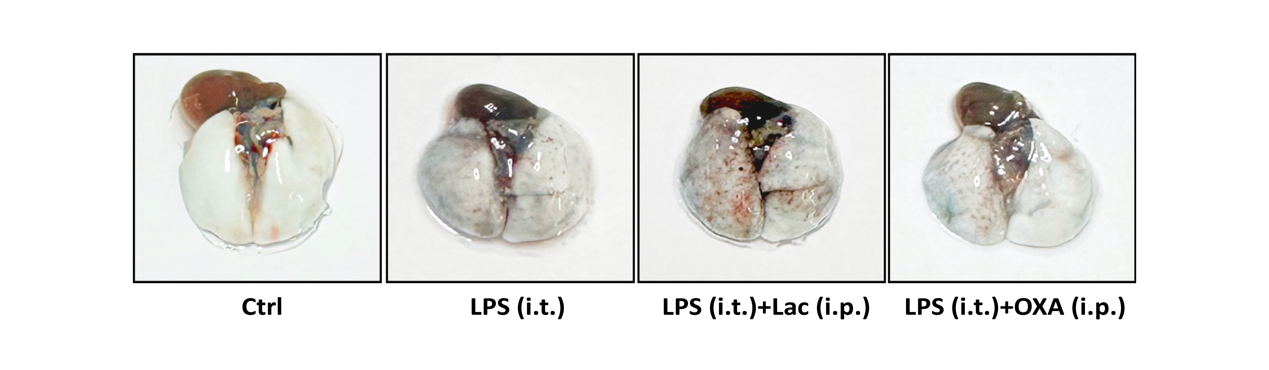


**Figure S2 Morphology of** **the lungs of mice in different groups.**

**
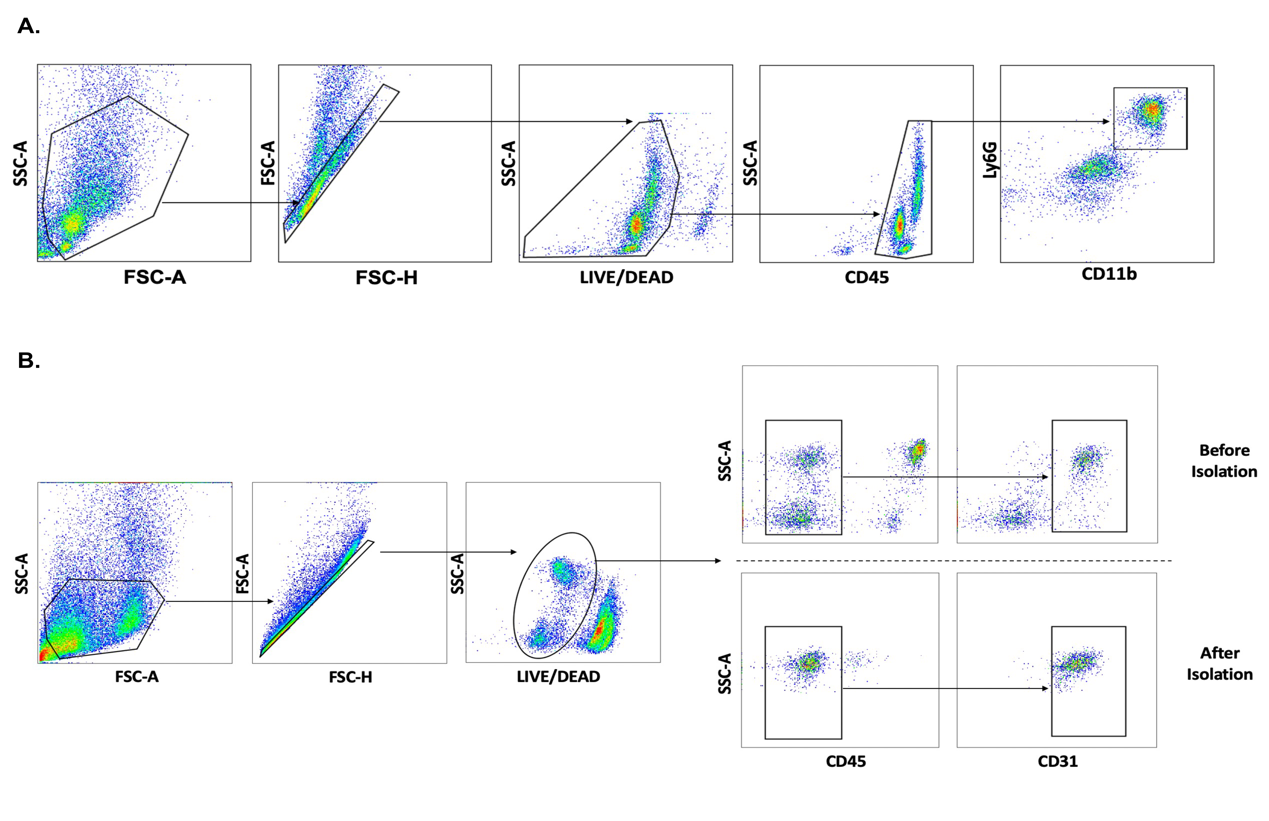
**

**Figure S3 Gate strategies** for (A) neutrophils in BALF and (B) isolated primary PECs.

**
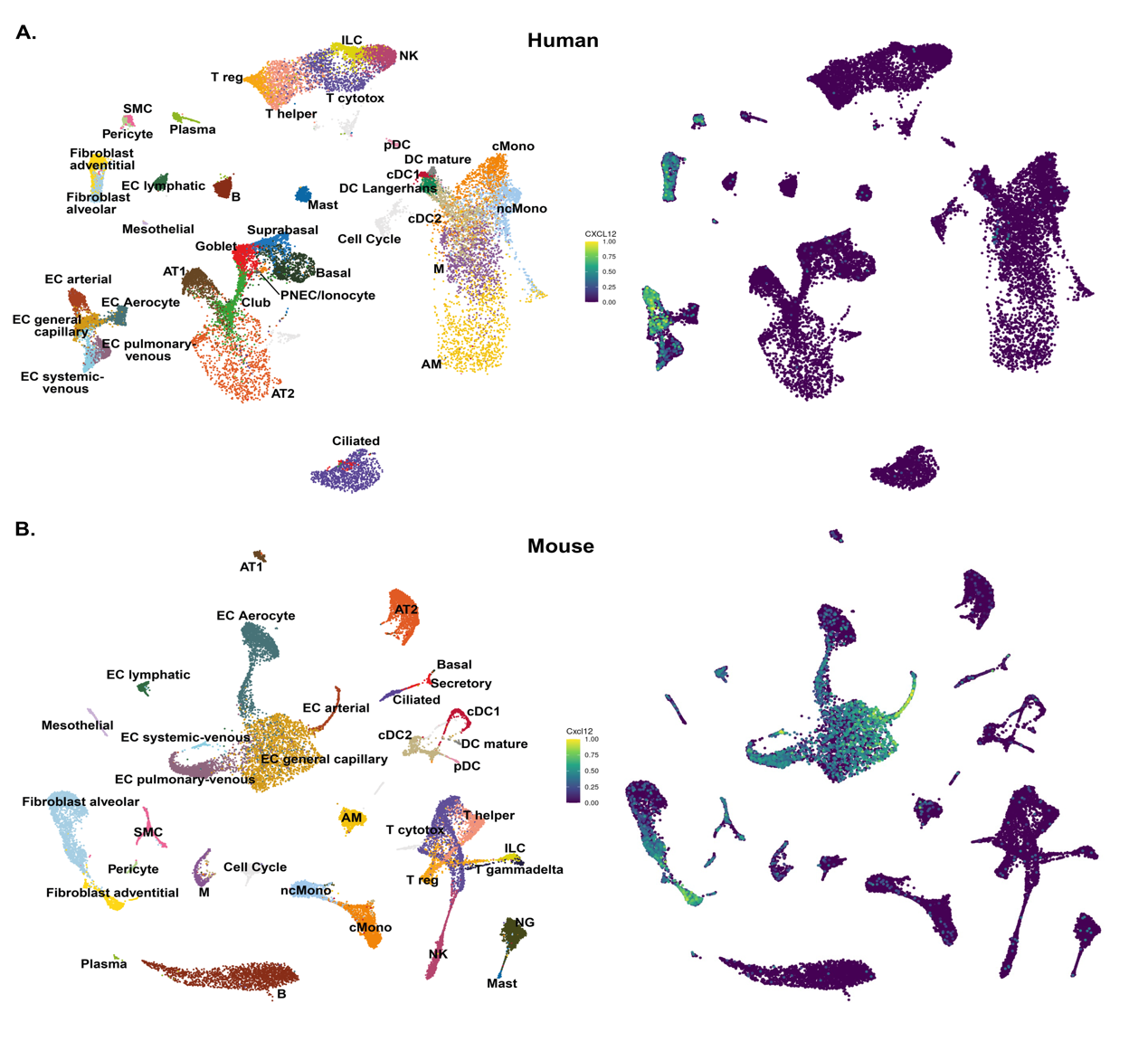
**

**Figure S4 Uniform Manifold Approximation and Projection (UMAP) plot depicting the expression of CXCL12 in the different cell populations in the mouse and human lungs.** CXCL12 is mainly expressed in pulmonary ECs and fibroblasts. This analysis was conducted using the Lung Endothelial Cell Atlas mining website (http://www.lungendothelialcellatlas.com).

**
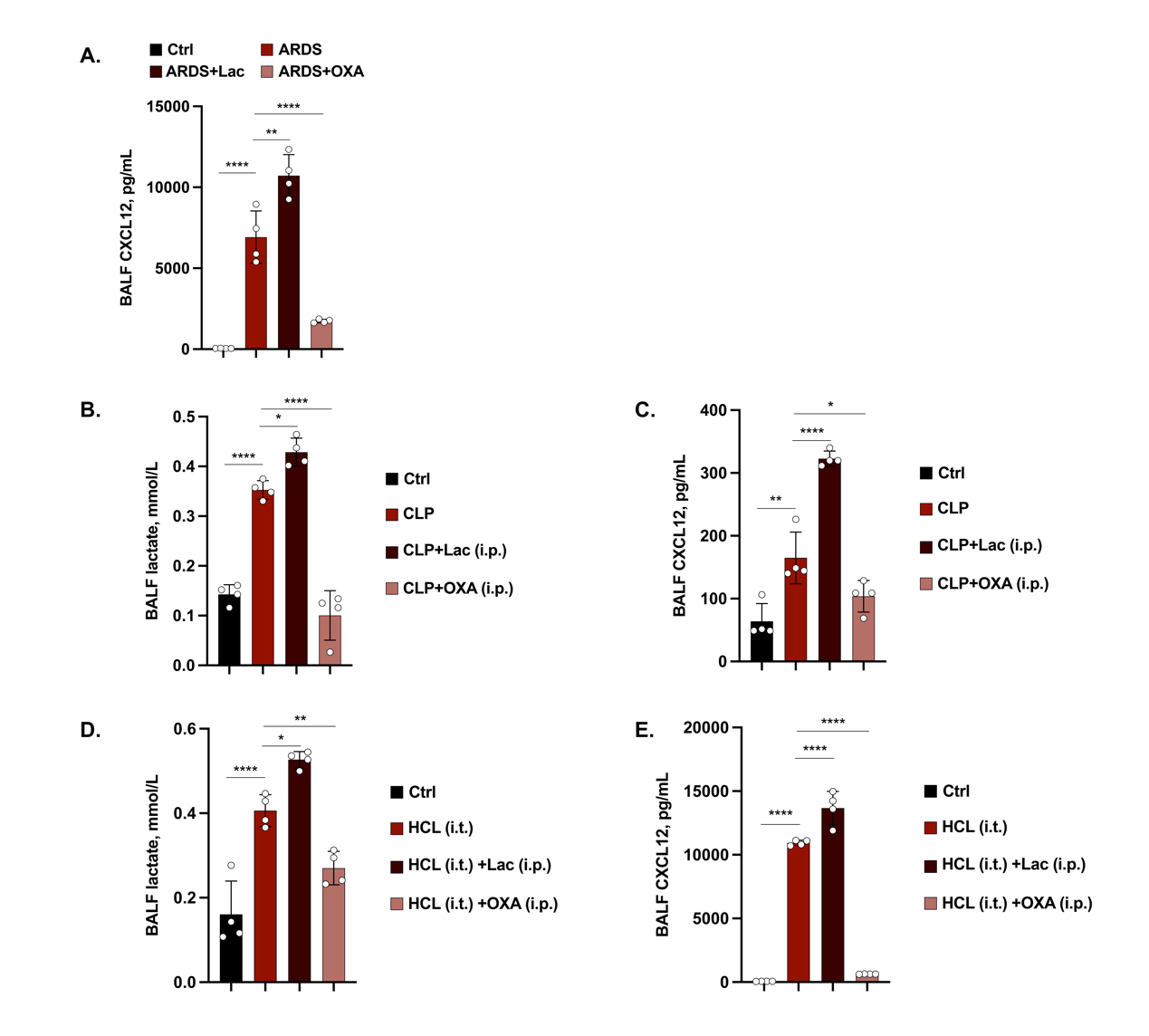
**

**Figure S5 Concentrations of CXCL12 in the BALF of ARDS mice were related to lactate levels.** (A) CXCL12 concentrations in the BALF of the control mice, LPS-induced ARDS mice, LPS-induced ARDS mice+Lac, and LPS-induced ARDS mice+OXA groups (n=4 per group). (B) Lactate levels in the BALF of the control mice, CLP mice, CLP mice+Lac, and CLP mice+OXA groups (n=4 per group). (C) CXCL12 concentrations in the BALF of the control mice, CLP mice, CLP mice+Lac, and CLP mice+OXA groups (n=4 per group). (D) Lactate levels in the BALF of the control mice, HCL mice, HCL mice+Lac, and HCL mice+OXA groups (n=4 per group). (E) CXCL12 concentrations in the BALF of the control mice, HCL mice, HCL mice+Lac, and HCL mice+OXA groups (n=4 per group). **p*<0.05; ***p*<0.01; *****p*<0.001.

**
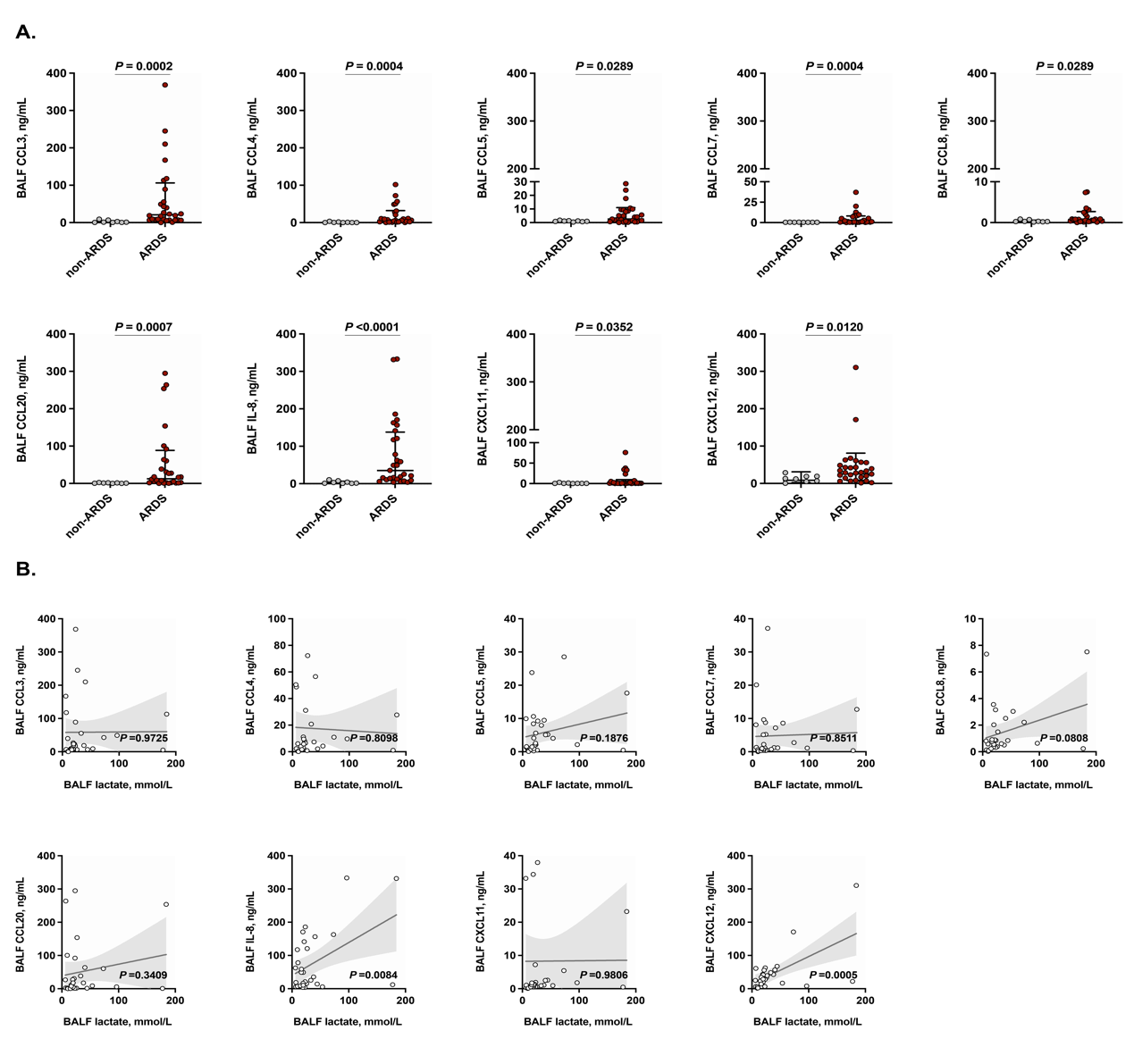
**

**Figure S6 Chemokine expression in the BALF of patients.** (A) The concentration of CCL3, CCL4, CCL5, CCL7, CCL8, CCL20, IL-8, CXCL11, and CXCL12 were elevated in the BALF samples of ARDS patients (n=29) compared with non-ARDS patients (n=8). (B) Correlation of BALF lactate with these chemokines in patients with ARDS.


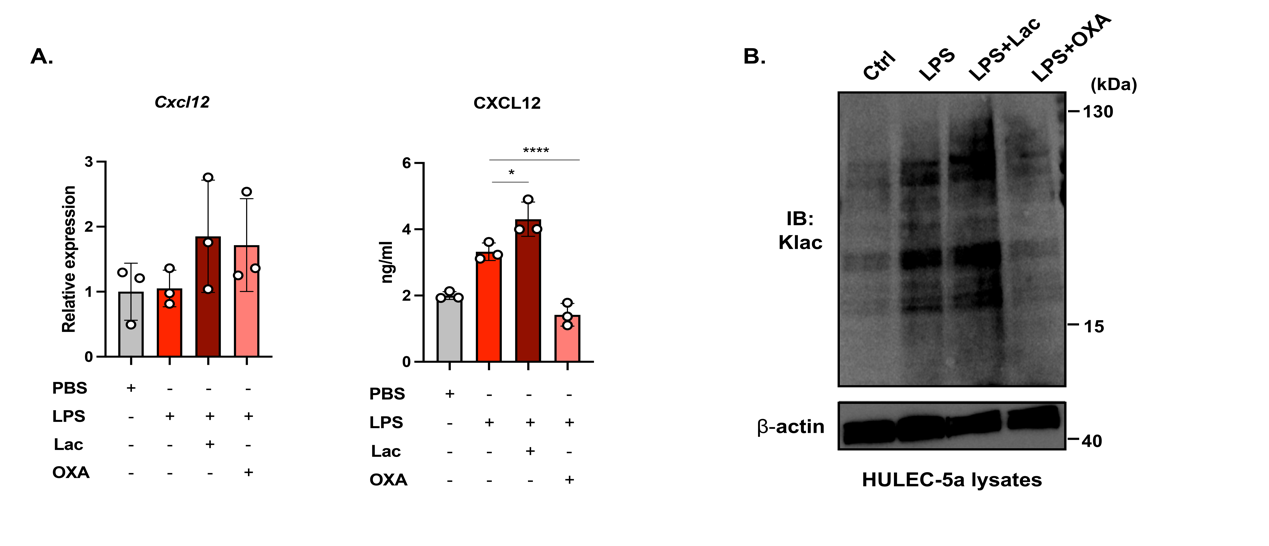


**Figure S7 The CXCL12 production in HULEC-5a following different treatments.** (A) The mRNA and protein levels of CXCL12 in HULEC-5a from the control, LPS, LPS+Lac, and LPS+OXA groups (n=3). (B) Western blotting of the pan-Klac levels in HULEC-5a from the control, LPS, LPS+Lac, and LPS+OXA groups. **p*<0.05; *****p*<0.001.

**
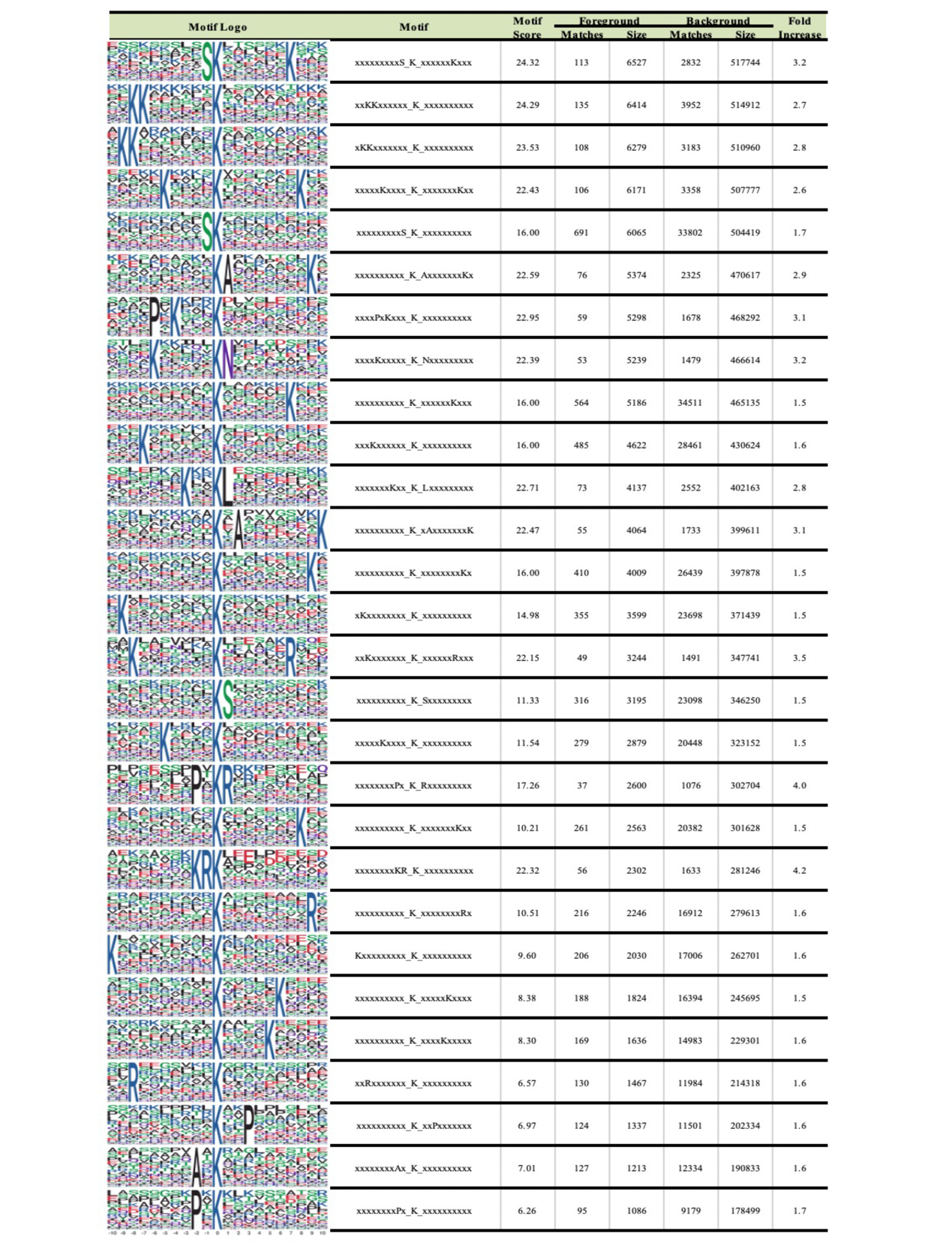
**

**Figure S8 Enriched motifs of Klac sites.**

**
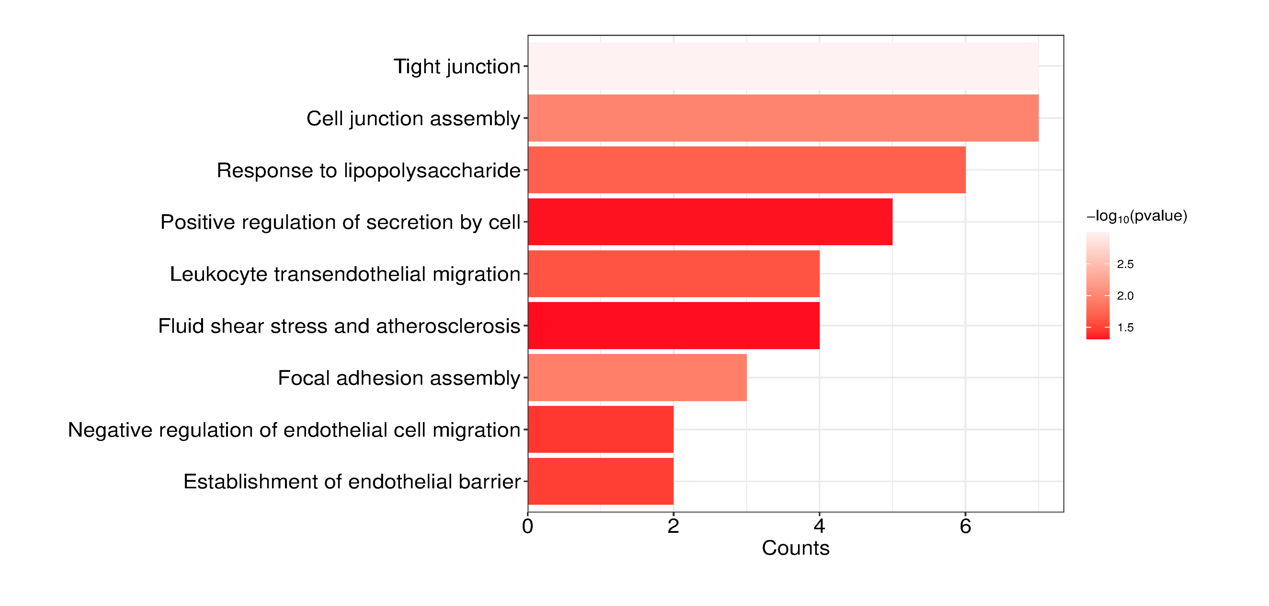
**

**Figure S9 Enriched endothelial-****associated biological processes of the** **Klac site (ARDS/Control>+∞) located proteins.**

**
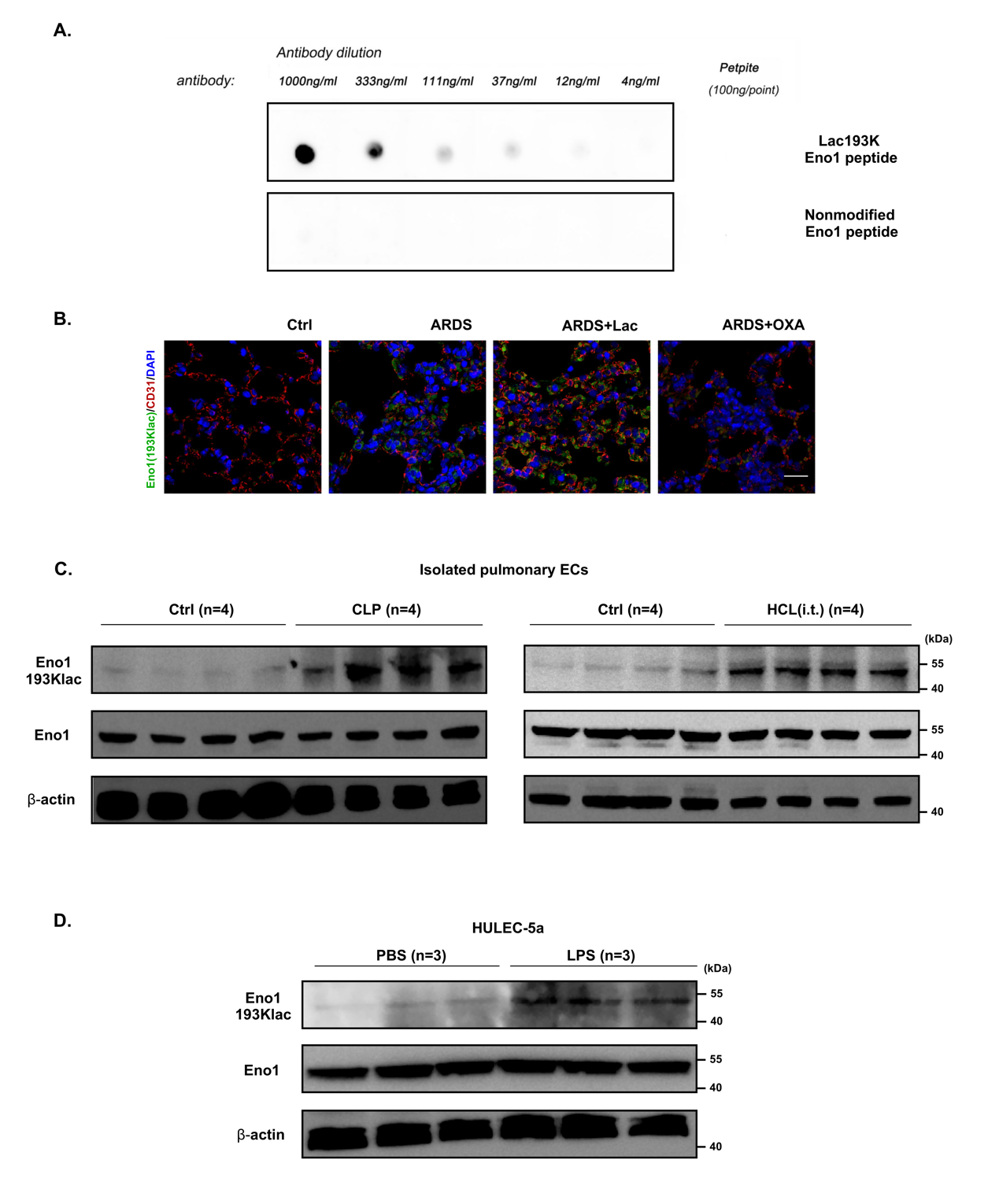
**

**Figure S10 K193 lactylation of Eno1 in the PECs** **of the ARDS mice and HULEC-5a cells.** (A) Dot plot assays using an antibody that detects Eno1 K193 lactylation. (B) Representative immunofluorescence image of K193 lactylated Eno1 in the PECs of control mice, LPS-induced ARDS mice, LPS-induced ARDS mice+Lac, and LPS-induced ARDS mice+OXA groups (n=4 per group). Scale bar, 10 µm. (C) Western blotting analysis of the K193 lactylated level in PECs isolated from the lungs of control mice and CLP-induced or HCL-induced ARDS mice. (D) *In vitro*, the K193 lactylation levels in PBS- and LPS-activated HULEC-5a were measured by western blotting.


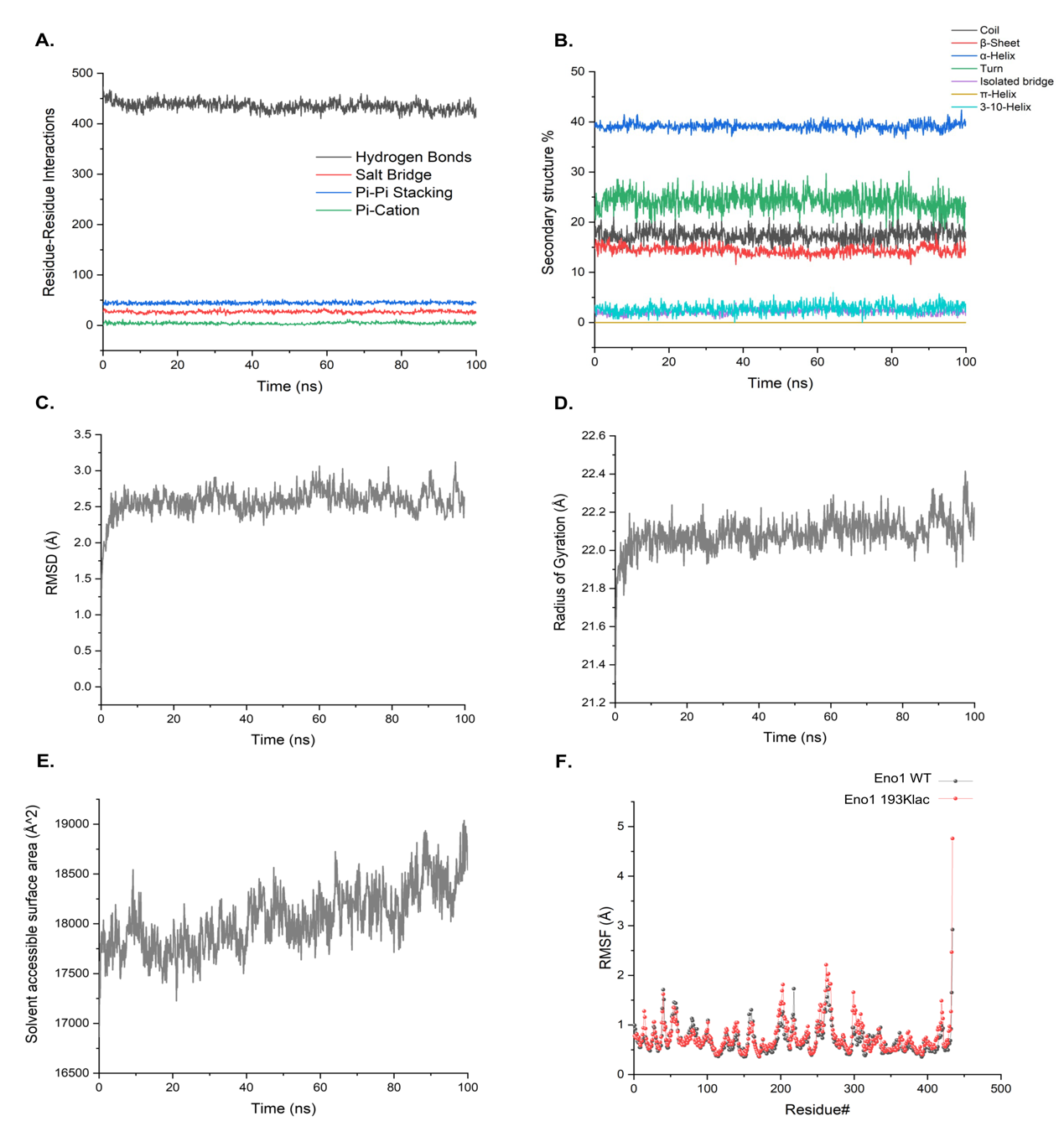


**Figure S11 K193 Lactylation on Eno1 changes its structure and characteristics.** (A) Statistics of WT and K193-lactylated Eno1 residue-residue interactions. (B) Statistics of WT and K193-lactylated Eno1 secondary structure content. (C-F) RMSD, solvent accessible area, radius of gyration and RMSF changes after K193 lactylation.


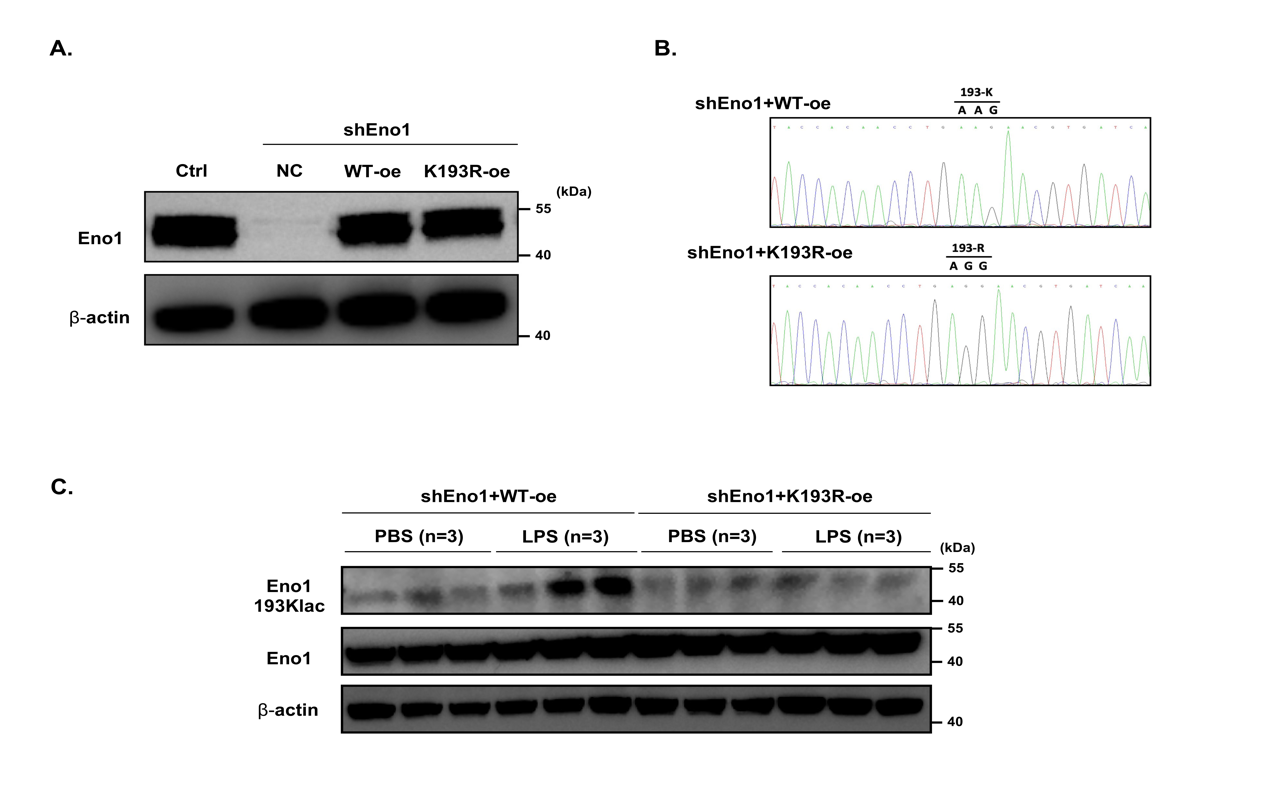


**Figure S12** **Lactylation levels of Eno1 in PMECs transfected with virus.** (A) Eno1 levels in MPMECs transfected with shEno1, shEno1+oe-WT, or shEno1+oe-K193R. (B) PCR combined with Sanger sequencing was used to identify whether 193 lysine was mutated to arginine (AAG to AGG). (C) Western blotting for Eno1 lactylation in PMECs in different groups.
